# The emerging role of calsequestrin 2: from calcium sensor and modulator to arrhythmia driver

**DOI:** 10.1007/s13105-026-01137-7

**Published:** 2026-02-02

**Authors:** Humam Emad Rajha, Baha H. Abuajameia, Ali Mohamed Barhoma, Ibrahim El-Arabi Hashem, Zeyaul Islam, Christopher Lai, F. Anthony Lai, Michail Nomikos

**Affiliations:** 1 College of Medicine, QU Health, Qatar University, Doha, Qatar; 2 Diabetes Research Center, Qatar Biomedical Research Institute (QBRI), Doha, Qatar; 3 Oxford University Clinical Academic Graduate School, John Radcliffe Hospital, Oxford, UK

**Keywords:** Calsequestrin 2, CASQ2, Arrhythmias, Catecholaminergic polymorphic ventricular tachycardia, CPVT

## Abstract

Calsequestrin 2 (CASQ2) has emerged as a central sensor and modulator of calcium (Ca^2+^) dynamics in sarcoplasmic reticulum (SR), influencing both health and disease. This review explores the molecular architecture and multifunctional roles of CASQ2, beginning with its domain organization and Ca^2+^-binding properties and detecting how its folding and supramolecular assembly modulate Ca^2+^ storage and release within cardiac muscle. Post-translational modifications, genetic regulatory mechanisms and CASQ2’s multipartner interactome; including Ryanodine receptor 2 (RyR2), triadin and junctin are also discussed to highlight potential models in which complex stoichiometry and luminal Ca^2+^ dictate channel refractoriness and excitation-contraction coupling. Disruption of CASQ2 function is increasingly recognized as a driver of certain types of arrhythmias, notably catecholaminergic polymorphic ventricular tachycardia (CPVT) and heightened risk of sudden cardiac death. This review appraises contemporary therapies that focus on pharmacological and device-based interventions and surveys next-generation strategies that aim to directly stabilize CASQ2 or target its gene expression. Despite therapeutic advances, the challenges remain; and a translational agenda aligning mechanism with therapy is proposed. By integrating recent structural, functional, regulatory and pathological insights, this review provides a conceptual framework for the pivotal role of CASQ2 in arrhythmogenesis and positions CASQ2 biology at the center of precision cardiology.

## Key points

CASQ2 regulates Ca^2+^ storage and release in cardiac muscle through its structure and binding properties.

CASQ2 dysfunction contributes to arrhythmias like CPVT and sudden cardiac death.

New therapies aim to stabilize or modulate CASQ2 for precision heart treatments.

## Introduction

Few elements are as vital to human life as calcium (Ca²⁺), a mineral whose influence reaches every corner of human physiology [1]. Beyond its well-known function in fortifying bones and teeth, Ca²⁺ is crucial for critical activities in the muscles, kidneys, and the nervous system. Meanwhile, in soft tissues, Ca²⁺ serves as a messenger, regulating essential processes such as cellular signaling, blood clotting, and hormone secretion [2].

However, the most dynamic role of Ca²⁺ is possibly in muscle physiology, as fluctuations in intracellular Ca²⁺ dictate the contraction and relaxation of muscle fibers [3]. A delicate balance in intracellular Ca²⁺ levels ensures the synchronization of systole and diastole, directly affecting cardiac output [4]. Therefore, disruptions in Ca²⁺ homeostasis can trigger a cascade of pathological events, leading to arrhythmias and cardiomyopathies [5].

In muscle cells, the intricate storage and dynamic release of Ca²⁺ are tightly regulated by the sarcoplasmic reticulum (SR), a specialized intracellular organelle that serves as the primary reservoir for Ca²⁺ [6]. Within the SR, Ca²⁺-binding proteins, such as calsequestrin (CASQ), act as the guardians of this reservoir, ensuring precise control over Ca²⁺ availability. Notably, CASQ is the most abundant Ca²⁺-binding protein localized in the SR of skeletal and cardiac muscle [7].

The history of CASQ began in 1971, when MacLennan and Wong first identified CASQ in rabbit skeletal muscle tissues [8, 9]. Consequently, this discovery laid the foundation for further studies into Ca²⁺-binding proteins, and a few years later, CASQ2 was identified as the cardiac isoform of CASQ, highlighting its important role in cardiac and slow-twitch muscle fibers [10].

Vertebrates’ genome contains two closely related genes, CASQ1 and CASQ2, which exhibit notable homology but distinct expression patterns. CASQ1 is exclusively expressed in fast-twitch skeletal muscle fibers, while both CASQ1 and CASQ2 are found in slow-twitch skeletal muscle fibers. Remarkably, cardiomyocytes express only CASQ2, which is uniquely adapted to the heart’s specific needs for precise and rhythmic Ca²⁺ handling and contraction [7, 11].

As the sole CASQ isoform within cardiac muscle, CASQ2 is fundamental to Ca²⁺ buffering and regulation within the SR. Given the heart’s high demand for rapid and coordinated contraction, the precise regulation of Ca²⁺ by CASQ2 is indispensable for maintaining normal cardiac rhythm and function [12].

Forming a quaternary complex with triadin, junctin, and ryanodine receptor 2 (RyR2), CASQ2 facilitates accurate communication between luminal Ca²⁺ levels and Ca²⁺ release channels. This interaction ensures tightly regulated Ca²⁺-induced Ca²⁺ release (CICR) during cardiomyocyte excitation-contraction (EC) coupling, thereby ensuring essential rapid and synchronized contractions of cardiac muscle. Additionally, CASQ2’s structural features, including its thioredoxin-like domains and acidic C-terminal tail, allow it to dynamically respond to changes in SR Ca²⁺ levels while maintaining the balance necessary for optimal cardiac function [12].

Building on these foundational insights into CASQ2’s structure and function, further breakthroughs in the late 1990 s and early 2000 s revealed the significance of CASQ2. A notable advancement came in 1998 when the first atomic resolution crystallographic structure of CASQ2 was published, providing insights into its Ca²⁺-binding mechanism and polymeric structure [13]. Subsequently, the association of CASQ2 with catecholaminergic polymorphic ventricular tachycardia (CPVT), a form of arrhythmogenic cardiac disease, was first established in 2001 through the discovery of missense variants in a family with autosomal recessive CPVT. In the following year, nonsense variants were identified in another family with a similar condition. Consequently, this was a pivotal moment in connecting CASQ2 dysfunction to cardiac arrhythmias [14].

Following these landmark discoveries, this review aims to provide a comprehensive update on CASQ2’s central role in cardiac muscle physiology, elucidating and emphasizing its structure, function, regulation, and interactions in cardiac muscle. It delves into the pathological consequences of CASQ2 dysfunction, linking it to arrhythmogenic cardiac diseases such as CPVT. When specific studies on CASQ2 or its related diseases are not available, this review attempts to extrapolate, associate and link findings from CASQ1 or CASQ studies in general under the implicit understanding that many molecular and pathophysiological mechanisms are to a large extent conserved. Furthermore, this review also highlights recent advancements in understanding CASQ2’s role and discusses emerging potential therapeutic approaches targeting its pathways, striving to inspire further research into its function and innovative approaches to address CASQ2-associated cardiac disorders. This review positions CASQ2 as not just a protein of interest but as a cornerstone in cardiac medicine.

## Structure

### Structural and functional overview of CASQ2 in cardiac muscle

An essential feature of efficient Ca^2+^ handling in cardiac muscle is the rapid availability of Ca²⁺ on demand, which is supported by the highly acidic CASQ2 protein and its coordinated interaction with key components of the Ca^2+^ release unit - junctin, triadin, and the RyR2 [15–17]. Despite its modest size (~ 45 kDa), CASQ2 is the most abundantly expressed protein within the lumen of the junctional sarcoplasmic reticulum (jSR), with concentrations reaching up to 100 mg/mL [9].

The *CASQ2* gene is situated on chromosome 1, at locus p13.3-p11, and is made up of 11 exons [18, 19]. The translated human CASQ2 protein comprises 399 amino acids. However, the initial 19 amino acids form a signal peptide sequence that is likely removed before CASQ2 reaches its final location—the jSR [20–22]. Although the length of the full CASQ2 protein may differ between species, there is a high level of similarity (approximately 89–96%) that is maintained among mammalian species [23].

### Domain organization and Ca^2+^-binding properties

**Fig. 1:**
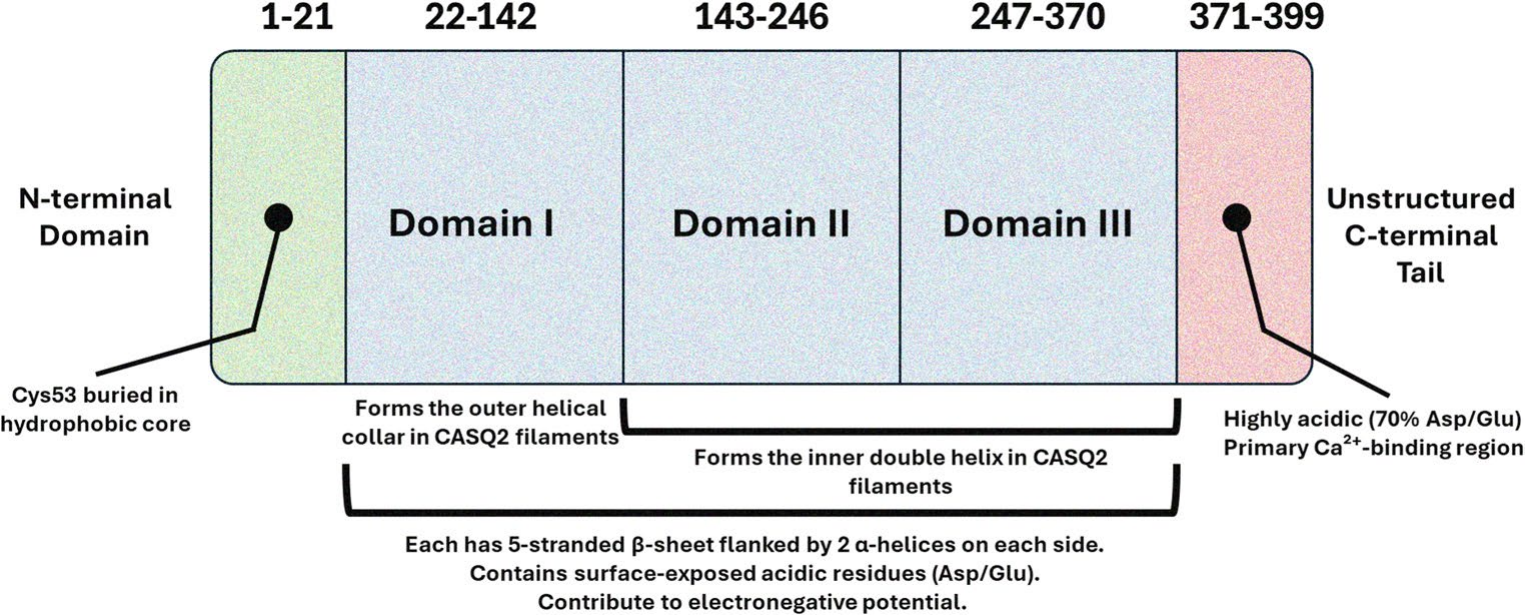
Domain organization of calsequestrin 2 (CASQ2). Structural architecture of the CASQ2 protein, which is composed of three distinct acidic thioredoxin-like (TrxL) domains (Domain I, Domain II, Domain III) and an unstructured C-terminal tail. The domains are arranged in a compact, globular structure, with each domain characterized by a β-sheet flanked by α-helices. The figure highlights the flexibility of CASQ2, enabling it to bind Ca^2+^ and undergo polymerization, which is critical for Ca^2+^ buffering in cardiac muscle cells. The N-terminal and C-terminal regions, marked by their acidic residues, are key in the protein’s functionality, contributing to its role in Ca^2+^ storage and release within the jSR

The quaternary structure of the compact, globular CASQ2 protein is composed of five distinct regions (Fig. 1): a brief N-terminal segment (amino-terminal loop), three acidic thioredoxin-like (TrxL) domains (referred to as domains I, II, and III), and an unstructured, flexible, and acidic C-terminal tail [21]. This architecture is reminiscent of that found in the endoplasmic reticulum (ER) luminal oxidoreductases [21]. The large number of acidic residues are distributed on the surface, particularly within loops and turns of the structure, facilitating high-capacity Ca^2+^-binding. Thus, maintaining the structural integrity of each region is essential for CASQ2’s ability to bind Ca²⁺ and to undergo polymerization given that the tri-domain structure allows for flexibility and extensive surface area necessary for CASQ2’s role in Ca^2+^ buffering [24].

The three TrxL domains (which make the overall structure of human CASQ2) are nearly identical in architecture. Each domain is composed of approximately 100 amino acids and features a five-stranded β-sheet flanked on both sides by two α-helices [21]. These three domains adopt a globular conformation, each exhibiting the conserved architecture of a hydrophobic core surrounded by β-sheets and α-helices. The domains span the following residue ranges: domain I (residues 22–142), domain II (143–246), and domain III (247–370). The conserved hydrophobic core and surface-exposed acidic residues contribute to the generation of the strong electronegative potential on the protein surfaces [21].

**Fig. 2:**
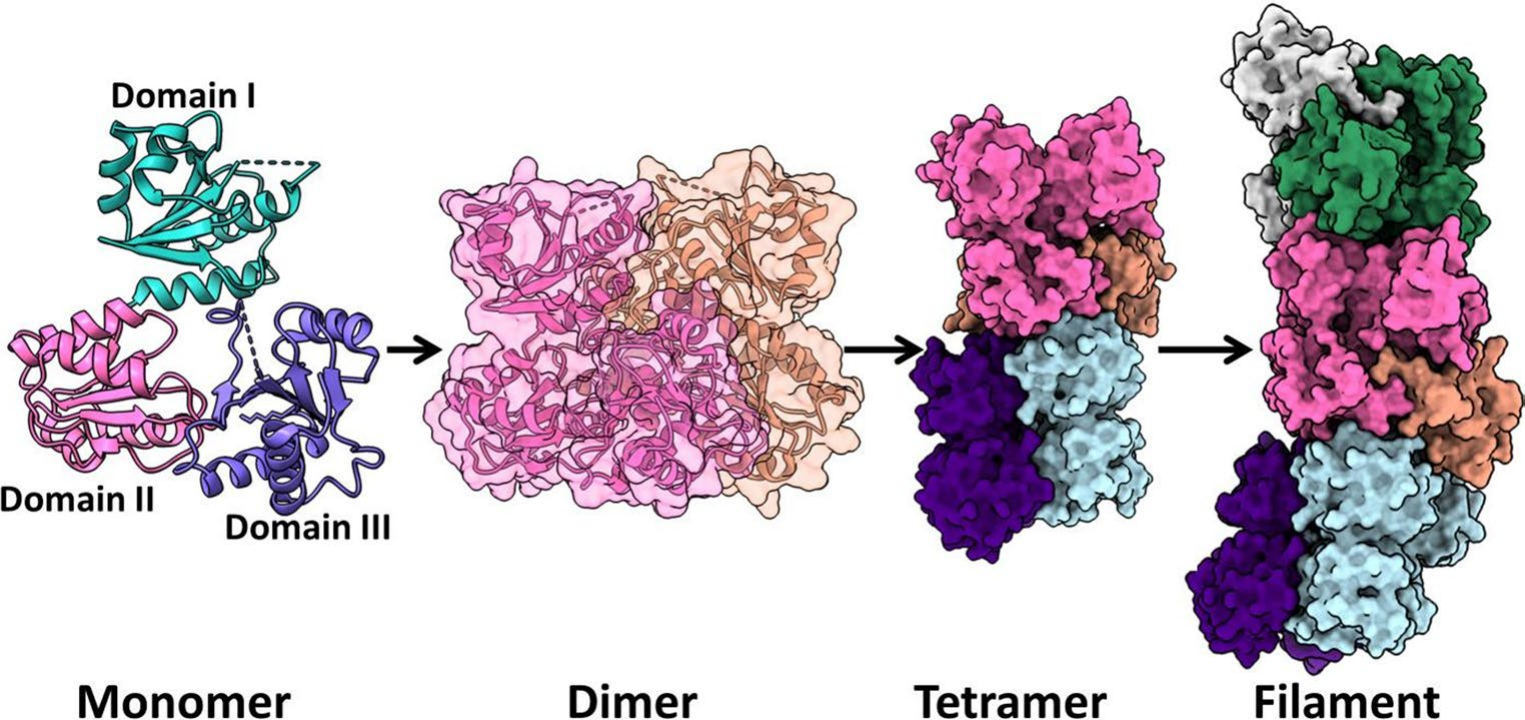
Calsequestrin 2 (CASQ2) higher order multimerization into filaments. Structure of several conformational states of CASQ2. The protein exists as a monomer and a wide range of higher order multimers. Cartoon as well as surface representation of CASQ2 monomer, dimer, tetramer and filament (PDBID: 6OWV). The CASQ2 monomer consists of three thioredoxin domains (domain I, II and III represented in green, pink and blue respectively). The higher multimer forming contacts exist between dimers and stacks along the screw axis to form the filament

Several structural studies using crystallography, size-exclusion chromatography, cross-linking and light scattering have highlighted the Ca^2+^-dependent dynamic polymers [25–27]. CASQ2 exists in multiple conformational states resulting in self-assembled higher oligomeric forms including filaments (Fig. 2). Ca^2+^-dependent transition from monomer to dimer has been reported which eventually led to inter-dimer interaction and higher-order multimer [28, 29]. The dimer stacks along a screw axis to create a filament where each dimer is rotated 90° relative to its neighbours. In the filament, CASQ2 structure exhibits a helical architecture with outer and inner helices formed by different thioredoxin domains [29].

The full functional roles of the TrxL domains are not yet fully understood. However, due to the presence of this conserved protein fold, both CASQ1 and CASQ2 are grouped within a large TrxL protein superfamily comprising over 4,000 members, all characterized by the presence of a TrxL domain. The TrxL superfamily also encompasses the protein disulfide isomerase (PDI) family [30, 31]. Despite their conserved tertiary structure, the proteins within the superfamily exhibit less than 30% similarity at the primary sequence level. Only a few of the proteins in this superfamily have been functionally characterized [30, 31].

Both CASQ isoforms lack the canonical C-x-x-C (cysteine-x-x-cysteine) catalytic motif typically required for thioredoxin enzymatic function [31]. Notably, the N-terminus of CASQ2—but not CASQ1—contains two highly conserved cysteine residues. However, their locations suggest they are unlikely to contribute to isomerase activity [30, 31]. Cys17 is part of the signal peptide, which is cleaved during protein synthesis and thus absent in the mature protein. Cys53 is located deep within the hydrophobic core of domain I’s thioredoxin-like fold, rendering it inaccessible for interaction with substrate proteins [30, 32].

The C-terminal domain of CASQ, rich in aspartic and glutamic acid residues, serves as the primary Ca^2+^-binding region on the monomer [33]. An extended C-terminal end contains more than 70% of acidic residues. Therefore, removing this domain from CASQ proteins leads to a reduction of over 50% in their Ca^2+^-binding capacity [33]. Notably, CASQ2 has a longer, more negatively charged C-terminus than CASQ1, contributing to the protein’s solubility and Ca^2+^-binding capacity as well as anchoring it to the jSR membrane [34].

### Ca^2+^-dependent folding, polymerization and supramolecular organization

CASQ2 has a dynamic and reversible structure; at low Ca^2+^ levels, CASQ2 exists in a molten globule state, then folds into compact monomers as Ca²⁺ rises [13, 16]. These monomers dimerize through N-terminal (front-to-front) interactions and further polymerize via C-terminal (back-to-back) stacking [24, 35]. Polymerization enhances CASQ2’s Ca^2+^-buffering capacity by forming negatively charged pockets that attract and bind Ca²⁺ ions [21, 36].

As for CASQ’s secondary and tertiary folding which is maintained by cations, all CASQ isoforms are rich in carboxylate groups from the aspartic and glutamic acid residues, giving human skeletal and cardiac CASQ isoelectric points of 4.0 and 4.2, respectively. At low ionic strength (below 100 mM KCl), electrostatic repulsion between these negative charges causes the protein to adopt an extended, random coil conformation [37, 38]. Multiple, monovalent, or divalent cations also play a role in directing the folding of CASQ’s highly similar, negatively charged TrxL domains where several hydrophobic interactions support the interior of the domains [39, 40]. The lowest ionic concentration necessary for maintaining CASQ’s secondary and tertiary folding depends on the coordination number and ionic radius of the cation. Among various cations, Ca²⁺ is the most efficient, showing effective binding at low concentrations and exhibiting notable cooperativity even under modest ionic conditions [41, 42].

In a study by Titus et al. (2020) to examine CASQ2’s filament structure, domain I forms an outer helical collar, while domains II and III form a compact inner double helix. The filament is built from stacked dimers rotated 90°, forming a left-handed helix with a continuous, electronegative lumen. This arrangement and binding site mapping were revealed under low pH crystallization conditions, enabling visualization of biologically relevant multimeric assembly for the first time [36].

## Function

CASQ2 has a variety of primary functions which include Ca²⁺ storage and buffering, regulation of Ca²⁺ release, EC coupling, maintenance of Ca²⁺ homeostasis, and stress response modulation. These roles emphasize its importance in cardiac function and its association with various pathophysiological conditions.

### Ca²⁺ storage and buffering

CASQ2 plays a critical role in Ca²⁺ storage and buffering within the SR [43]. It binds approximately 40–60 moles of Ca²⁺ per mole of protein. CASQ2’s low affinity binding enables the SR to maintain a substantial reservoir of Ca²⁺ for rapid mobilization during muscle contraction [24]. At high luminal Ca²⁺ levels (> 1 mM), CASQ2 polymerizes into filamentous structures, enhancing its Ca²⁺-binding capacity [24]. Conversely, at low Ca²⁺ levels, it depolymerizes, enabling the bound Ca²⁺ to be released into the cytosol to initiate contraction [24, 44].

As a reversible Ca^2+^ buffer, CASQ2 reduces free Ca²⁺ concentration within the SR, lowering the gradient against which the sarcoplasmic reticulum Ca²⁺-ATPase (SERCA) operates [45, 46]. This buffering action supports efficient Ca²⁺ reuptake and prevents cytotoxicity caused by elevated Ca²⁺ levels [47]. Dysregulation of CASQ2’s buffering capacity, observed in pathological conditions such as heart failure, leads to impaired Ca^2+^ homeostasis, contributing to arrhythmias and contractile dysfunction [48, 49].

### Regulation of Ca²⁺ release

CASQ2 directly interacts with RyRs, stabilizing these SR Ca²⁺ release channels and modulating their responsiveness to Ca²⁺. It acts as a sensor for luminal Ca²⁺ levels, promoting RyR opening at high Ca²⁺ concentrations and inhibiting it at low concentrations [50, 51]. This precise regulation ensures controlled Ca²⁺ dynamics during muscle contraction [52].

During CICR, a small influx of Ca²⁺ through voltage-gated Ca²⁺ channels activates RyRs [50, 53]. CASQ2 undergoes conformational changes upon Ca²⁺ binding, enhancing RyR opening and amplifying Ca²⁺ release from the SR [52]. This rapid increase in cytosolic Ca²⁺ triggers actin-myosin interactions, driving contraction. Mutations in CASQ2, such as R33Q, disrupt this interaction, leading to spontaneous Ca²⁺ release and arrhythmias, such as catecholaminergic polymorphic ventricular tachycardia (CPVT), particularly under stress [54–56].

### Excitation-Contraction (EC) coupling

In cardiac EC coupling, an action potential spreads over the sarcolemma, penetrates the T-tubules and causes activation of voltage-gated L-type Ca²⁺ channels (dihydropyridine receptors) [12]. The ensuing influx of a small amount of extracellular Ca²⁺ into the cytosol, which binds to the cytosolic region of RyR2 [57], serves as the spark for the massive release of Ca²⁺ from the SR through RyR2 channels, a process known as CICR [58].

CASQ2 acts as a high-capacity, moderate-affinity Ca²⁺ buffer within the SR lumen. It binds large amounts of Ca²⁺, thus maintaining a readily releasable pool of Ca²⁺ for subsequent contractions. By doing so, CASQ2 sustains the Ca²⁺ gradient across the SR membrane and helps ensure that sufficient Ca²⁺ is available when needed for contraction.

In addition to regulating release, CASQ2 supports efficient Ca²⁺ reuptake during muscle relaxation. As it buffers Ca²⁺ in the SR, it facilitates the activity of the SERCA pump, which transfers cytosolic Ca²⁺ back into the SR. By doing so, CASQ2 helps restore SR Ca²⁺ levels between heartbeats and prevents cytosolic Ca²⁺ overload, a condition that could otherwise promote delayed afterdepolarizations and trigger arrhythmias [59–61].

This finely tuned balance is especially crucial during conditions of increased cardiac demand, such as exercise or emotional stress, where heart rate and intracellular Ca²⁺ fluxes are elevated [4]. Under such circumstances, CASQ2’s role in stabilizing SR Ca²⁺ content becomes even more important for preserving the synchrony between electrical excitation and mechanical contraction.

### Maintenance of Ca²⁺ homeostasis

CASQ2 is an example of specialized evolutionary adaptation optimized for CICR in cardiac muscle [62]. Unlike CASQ1, which supports depolarization-induced Ca²⁺ release in skeletal muscle, CASQ2’s function ensures precise regulation of Ca²⁺ signaling dynamics in response to electrical activity [60]. It balances free and bound Ca²⁺ within the SR, maintaining free Ca²⁺ at approximately 1 mM while allowing total concentrations to reach up to 19 mM [51, 63]. This balancing act is crucial for Ca²⁺ reuptake and availability during contraction [64].

CASQ2 also modulates store-operated Ca²⁺ entry (SOCE), a pathway for replenishing SR Ca²⁺ stores post-depletion [65]. It interacts with stromal interaction molecule 1 (STIM1), an SR Ca²⁺ sensor, inhibiting SOCE through luminal Ca²⁺ stabilization. Loss of CASQ2 enhances SOCE activity, disrupting Ca²⁺ homeostasis and increasing susceptibility to arrhythmias [51, 66, 67].

In addition to Ca²⁺ binding, CASQ2 exhibits ion selectivity, interacting with divalent and monovalent cations [46]. While its physiological relevance is dominated by Ca²⁺ binding, interactions with other ions, such as cadmium or zinc, may influence its structure and function under pathological conditions [68].

### Stress response and protection

CASQ2 contributes significantly to the cardiac response to physiological and pathological stress. It interacts with the unfolded protein response (UPR) sensor Inositol-Requiring Enzyme 1 alpha (IRE1α), preventing its dimerization and subsequent UPR activation under normal conditions [60, 69]. Disruption of this interaction, as seen in CASQ2 mutations, exacerbates stress signaling pathways, leading to cardiomyopathy and arrhythmias [70]. By modulating stress responses, CASQ2 ensures efficient cardiac function even under adverse conditions [71].

CASQ2 also protects cardiac tissue from oxidative stress by minimizing Ca²⁺ overload-induced damage [72]. Pharmacological agents such as dantrolene, which enhance CASQ2 polymerization and buffering capacity, underscore its potential as a therapeutic target in stress-induced cardiac conditions [73]. Additionally, CASQ2’s role in maintaining rhythm stability by preventing spontaneous Ca²⁺ release is vital, particularly during elevated adrenergic stimulation [74]. Evidence from knockout animal models links CASQ2 dysfunction to sinoatrial node defects and arrhythmias such as atrial fibrillation (AF) [75].

### Developmental role

CASQ2 may also play a role in cardiac and skeletal muscle development. Its expression, regulated by transcription factors like MEF-2 C and pathways such as calcineurin/Nuclear Factor of Activated T-cells (NFAT), suggests its involvement in stabilizing Ca²⁺ signaling mechanisms during development [76–78].

CASQ2 knockout animals exhibit increased susceptibility to stress-induced cardiac dysfunction, highlighting the importance of CASQ2 in maintaining Ca²⁺ homeostasis during early development. Disrupted CASQ2 expression can impair myocyte maturation and predispose the heart to arrhythmias or cardiomyopathy, underscoring its role in establishing a stable Ca²⁺ environment during growth [79, 80].

## Regulation

### Phosphorylation

Phosphorylation of calsequestrin 2 plays an important role in its function and structural dynamics. CASQ2 gets phosphorylated on its C-tail by casein kinase 2 [12]. This enzyme acts on specific sites for phosphorylation, such as Ser385 and Ser393. These sites were identified as phosphorylation sites of CASQ2 by Park et al. (2005) using mass spectrometry-based phosphoproteomics [81]. Functionally, dual phosphorylation of these sites significantly enhance CASQ2 helical content, solubility, and Ca²⁺-binding capacity when Ca²⁺ levels exceeded 6 mM. Structural studies highlighted that these modifications alter the conformation and charge of CASQ2’s C-tail, modulating an important role in CASQ2 function. In contrast, phosphorylation of one site has no significant effect, highlighting the crucial role of dual site phosphorylation in modulating CASQ2 function [81].

Newly synthesized CASQ2 is fully phosphorylated; suggesting that phosphorylation is essential for its initial structural integrity and functionality. Furthermore, dephosphorylation of CASQ2 in cardiac tissue has been documented with regard to a constitutive phosphate turnover of CASQ2 in the heart, and this turnover may be associated with its intracellular transport and SR localization [82]. While direct functional data linking CASQ2 dephosphorylation to Ca²⁺-release during cardiac EC coupling remain limited, this finding emphasizes that a dynamic balance between phosphorylation and dephosphorylation is likely vital for CASQ2 structural integrity and function.

Phosphorylation of CASQ2 has been also shown to play a role in its protein transport and localization within the cell [12] by making CASQ2 highly soluble and compact. However, McFarland and colleagues (2010) found that the loss of CASQ2 phosphorylation enhances anterograde trafficking [83]. The co-translocational hypothesis resolves this discrepancy in the literature, suggesting that phosphorylation takes place in the cytoplasmic domain of CASQ2 during its translocation through the translocon into the ER, before it fully localizes to the lumen of the SR.

Another critical yet less-studied kinase involved in CASQ2 phosphorylation is Fam20C. Fam20C phosphorylates CASQ2 and other SR proteins, playing an essential role in regulating Ca²⁺ homeostasis and providing cardioprotection by preventing heart failure. Unlike casein kinase 2, which targets specific C-tail residues, Fam20C has broader regulatory effects on SR protein function, contributing to overall Ca²⁺ dynamics [84].

### Glycosylation

In addition to phosphorylation, another essential post-translational modification that calsequestrin undergoes is N-linked glycosylation. This modification significantly influences its structure, stability, and function [23]. This process occurs co-translationally in the ER and involves the enzymatic activity of the oligo-saccharyl transferase complex [85]. This enzyme transfers a pre-assembled glycan moiety to specific asparagine residues within the consensus motif N-X-S/T of CASQ during its synthesis and translocation into the ER lumen. Human CASQ2 has two known glycosylation sites, N212 and N335 [85]. Glycosylation at these sites stabilizes the tertiary structure of CASQ, particularly Domain III [86], which plays a vital role in monomer alignment during polymerization. The presence of oligosaccharides acts as a “molecular guiderail”, ensuring directing or stabilizing the proper formation and alignment, and the generation of stable and linear CASQ polymers [87].

Glycosylation not only enhances the solubility and Ca²⁺-binding efficiency of CASQ, it also enables it to respond effectively to changes in Ca²⁺ concentrations during EC coupling [88]. Furthermore, glycosylation promotes CASQ trafficking through the secretory pathway to its functional site at the SR junction (SRJ). Though this observation was originally reported for the skeletal muscle isoform CASQ1 [88], cardiac CASQ2 exhibits more extensive glycosylation, with CASQ2 having an average of six mannoses per glycosylation site compared to one mannose in CASQ1, reflecting its unique functional demands in cardiac tissue [23]. Disruptions in glycosylation, whether due to mutations or enzymatic deficiencies, can impair CASQ’s polymerization and Ca²⁺-buffering capacity, contributing to pathological conditions such as catecholaminergic polymorphic ventricular tachycardia (CPVT) [12].

### Genetic regulation

The transcription of the *CASQ2* gene, encoding the cardiac-specific isoform of calsequestrin, is tightly regulated by a combination of transcription factors, promoter architecture, and Ca^2+^-dependent signaling pathways. The Ca²⁺-calcineurin/NFAT pathway plays a central role in modulating CASQ2 expression in cardiomyocytes, where NFAT acts as a positive regulator - inhibition of NFAT dephosphorylation reduces CASQ2 mRNA levels [89]. Additionally, Myocyte Enhancer Factor-2 (MEF-2) binding sites within the *CASQ2* promoter are essential for transcriptional activation, and NFAT cooperates with MEF-2 to enhance gene expression [90]. This synergy highlights a Ca^2+^-sensitive mechanism where calcineurin-NFAT signaling, triggered by intracellular Ca²⁺, directly links cardiac activity to gene regulation.

The proximal promoter region of *CASQ2*, spanning approximately 180 base pairs upstream of the transcription start site, contains several evolutionarily conserved regulatory elements. These include a TATA box for transcription initiation, a CArG (CC(A/T)6GG) box (recognized by serum response factor (SRF) to drive muscle-specific expression), an E-box (targeted by myogenic regulators like MyoD and myogenin), and MEF-2 sites critical for muscle gene activation [91]. These motifs collectively ensure tissue-specific and developmentally appropriate CASQ2 expression, particularly in cardiac and slow skeletal muscle fibers. The interplay between SRF, MEF-2, and myogenic factors underscores the combinatorial control of *CASQ2* transcription, fine-tuning its levels to match functional demands.

Beyond transcriptional regulation, hormonal and epigenetic mechanisms may further influence CASQ2 expression. Thyroid hormone has been shown to modulate CASQ isoforms levels in non-mammalian models, suggesting potential conserved roles in vertebrates [92]. Epigenetic modifications, such as DNA methylation or histone acetylation, could also contribute to silencing CASQ2 in non-muscle tissues, though experimental validation in mammals is still needed. Together, these regulatory layers - transcription factor networks, promoter architecture, Ca^2+^ signaling, and secondary hormonal/epigenetic inputs - ensure precise CASQ2 expression, optimizing Ca^2+^ handling and contractile function in the heart.

## Protein interactions

### Interaction with ryanodine receptor 2

Cardiac CASQ2 interacts directly with the luminal domain of RyR2 at its first luminal loop, forming a crucial component of the SR Ca²⁺ sensor [11]. Structural studies reveal that this binding occurs through electrostatic interactions between acidic residues in CASQ2’s C-terminal dimerization domain and basic residues in RyR2’s luminal loop. This interaction, supported by accessory proteins triadin and junctin, modulates RyR2 function and SR Ca²⁺ release [93]. Together, these proteins form the " Ca^2+^- release complex”, where triadin and junctin act as molecular scaffolds that enhance the physical and functional coupling between CASQ2 and RyR2.

**Fig. 3:**
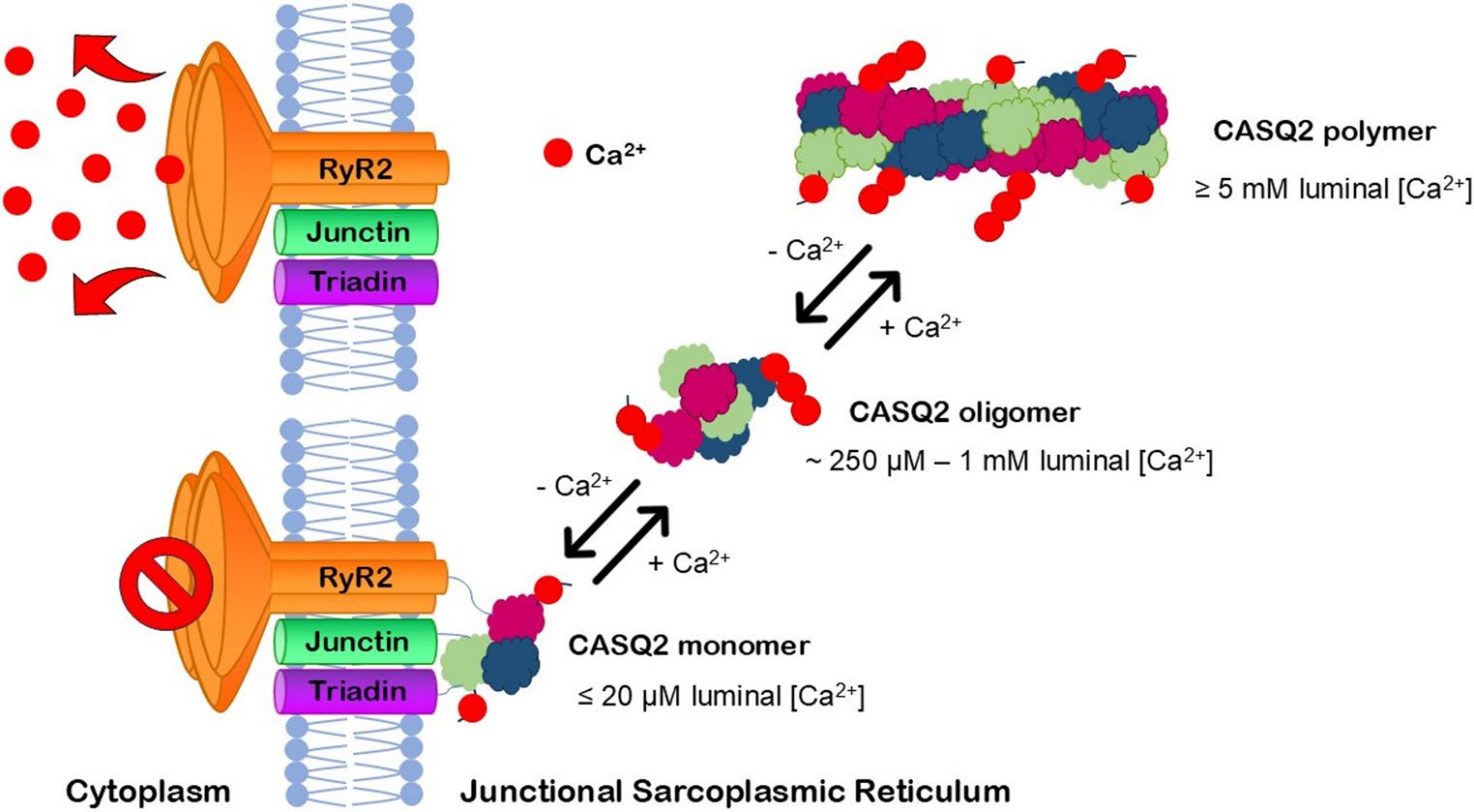
**Ca**^**2+**^**-Dependent Regulation of RyR2 by CASQ2 within the Junctional Sarcoplasmic Reticulum (jSR) of Cardiac Muscle.** At high luminal Ca²⁺ concentrations (≥ 5 mM), CASQ2 has the lowest affinity for RyR2, polymerizes in the SR lumen enhancing Ca²⁺ storage and has no inhibitory effect on RyR2. As luminal Ca²⁺ decreases (~ 250 µM − 1 mM), CASQ2 depolymerizes into small oligomers with intermediate affinity for RyR2 and minimal regulatory effect on the Ca²⁺ channel. When Ca²⁺ levels fall to ≤ 20 µM, monomeric CASQ2 has the highest affinity for the RyR2 and effectively inhibits RyR2 channel activity via interactions with triadin and junctin, preventing spontaneous Ca²⁺ release. This reversible mechanism ensures controlled Ca²⁺ dynamics critical for cardiac contraction and relaxation

CASQ2 serves as both a Ca²⁺ storage site and a luminal Ca²⁺ sensor, inhibiting RyR2 opening at low luminal Ca²⁺ concentrations and regulating Ca²⁺ release. At low Ca²⁺ levels (< 1 mM), monomeric CASQ2 binds tightly to RyR2, suppressing channel activity to prevent diastolic Ca²⁺ leaks. The CASQ2–RyR2 association is Ca²⁺-dependent; monomeric CASQ2 at low luminal Ca²⁺ inhibits RyR2, whereas polymerization at higher Ca²⁺ (> 5 mM) diminishes this inhibition [45]. This polymerization triggers conformational changes that reduce CASQ2’s affinity for RyR2, effectively releasing inhibition and priming the channel for systolic Ca²⁺ release. Figure 3 illustrates how CASQ2 regulates Ca^2+^ release through its interaction with RyR2.

Recent cryo-EM studies have mapped the precise binding interface between CASQ2 and RyR2, showing that disruption of this interaction leads to aberrant Ca²⁺ sparks and arrhythmias in cardiomyocytes [11]. Furthermore, mutations in either CASQ2 or RyR2 that impair their co-binding are linked to CPVT, underscoring the physiological importance of this interaction [94].

### CASQ2 interaction with triadin

CASQ2 and triadin are key components of the junctional SR complex, critical for Ca²⁺ release regulation. Triadin is a transmembrane protein that connects CASQ2 to RyR2, forming a structural scaffold that stabilizes the Ca²⁺ release unit [60]. CASQ2 modulates RyR2 activity by inhibiting premature Ca²⁺ release through its association with triadin [7]. Disrupting this interaction increases the sensitivity of SR Ca²⁺ release to triggers, causing spontaneous Ca²⁺ oscillations and arrhythmic membrane depolarizations. The interaction also supports SR morphology; triadin ablation reduces junctional SR extent by 50%, impairing Ca²⁺ release dynamics [60]. Isoform-specific effects further refine regulation, with triadin-1 predominating in cardiac tissue and exhibiting distinct roles in RyR2 gating. Loss of triadin disrupts CASQ2-RyR2 coupling, increasing susceptibility to stress-induced arrhythmias. The binding between CASQ2 and triadin is mediated by specific amino acid motifs, triadin contains Lysine (K)–Glutamate (E)–Lysine (K)–Glutamate (E) (KEKE) motifs in its luminal domain, which are responsible for binding to CASQ2. This interaction is essential for anchoring CASQ2 to the junctional face membrane, allowing it to sequester Ca²⁺ in the vicinity of the RyR2 during Ca²⁺ uptake and release [50]. The aspartate-rich region of CASQ2 also plays a role in this interaction, providing a direct binding site for both luminal Ca²⁺ and triadin [95].

### CASQ2 interaction with junctin

Junctin is an integral component of the SR Ca²⁺ release complex, directly interacting with CASQ2, triadin and RyR2 [60]. Like triadin, the junctin luminal domain is rich in charged amino acids and KEKE motifs, and this domain binds CASQ2 and stabilizes the quaternary complex [96]. Junctin facilitates the localization of CASQ2 to the junctional SR, ensuring its proper association with RyR2 for efficient Ca²⁺ release regulation [50]. CASQ2’s Ca²⁺-dependent polymerization and depolymerization cycles require junctin, highlighting its role in maintaining CASQ2’s scaffold-like structure. The CASQ2-junctin interaction is essential for modulating RyR2 gating and buffering SR Ca²⁺ [45]. Overexpression of junctin disrupts Ca²⁺ signaling by altering the organization of Ca²⁺ release units, while mutations affecting junctin’s stability impair Ca²⁺ handling and contribute to cardiac hypertrophy and contractile dysfunction.

### CASQ2 and its synergistic role in the sarcoplasmic reticulum protein complex

CASQ2, along with triadin and junctin, forms a quaternary complex with RyR2. This complex is necessary for the normal operation of Ca²⁺ release in cardiac muscle. The KEKE motifs in triadin and junctin facilitate the binding of CASQ2 to RyR2, stabilizing the complex and ensuring efficient Ca²⁺ signaling [96]. The complex functions as an integrated system to facilitate Ca²⁺ storage and release in response to fluctuating luminal Ca²⁺ levels. At low SR Ca²⁺, CASQ2 remains monomeric and exerts an inhibitory effect on RyR2 via triadin/junctin. As Ca²⁺ levels rise, CASQ2 polymerizes into a filamentous matrix, weakening its interaction with RyR2 and relieving channel inhibition to permit Ca²⁺ release [51]. Disruption in this synergistic network, whether through mutations in CASQ2, triadin deficiencies, or junctin imbalances, leads to impaired Ca²⁺ homeostasis, triggering arrhythmogenic disorders and contractile dysfunction. These interactions demonstrate the delicate balance required for precise Ca²⁺ regulation in cardiac muscle [15, 93].

## Pathology

CASQ2 is undoubtedly a key protein in muscular functions; without it, Ca²⁺ handling and coordination would be impaired, leading to severe pathological consequences. Any dysregulation in CASQ2 normal functioning often leads to arrhythmogenic events.

Cardiac arrhythmias are disturbances in the normal rhythm of the heart, arising from abnormalities in impulse generation or conduction. They can be broadly classified into acquired and inherited types. Acquired arrhythmias are typically caused by external factors like drugs or underlying diseases [97–101], while inherited arrhythmias result from genetic mutations. Examples of inherited arrhythmias include CPVT and arrhythmogenic right ventricular cardiomyopathy (ARVC), while long QT syndrome can be either inherited or acquired. These conditions are frequently linked to mutations in SR proteins [102–104], leading to Ca²⁺ dysregulation that manifests through early afterdepolarizations (EADs), delayed afterdepolarizations (DADs), or reentrant circuits [105].

The dysfunction of CASQ2 is associated with various inherited cardiac conditions, most notably arrhythmias. This section explores the conditions linked to CASQ2 dysfunction, highlighting the underlying mutations and pathophysiological mechanisms.

### Catecholaminergic polymorphic ventricular tachycardia (CPVT)

The primary cardiac condition related to CASQ2 dysfunction is CPVT. The true prevalence of CPVT in the population is not known. An estimate of CPVT prevalence is 1:10,000 or less [106], with CASQ2-related CPVT2 accounting for about 5% of these cases [107]. Although much of the epidemiological and clinical data available in the literature describe CPVT in general, the underlying arrhythmogenic mechanism is shared across subtypes, namely, stress-induced SR Ca²⁺ leak leading to DADs.

Mutations such as R33Q, R35W, and E202K in the CASQ2 gene are commonly implicated in this CPVT2 subtype. CASQ2 mutations are often autosomal recessive and can manifest as missense, nonsense, or frameshift variants, leading to loss of CASQ2 protein functionality or stability [108]. The D307H and R33Q mutations are particularly well-documented, with studies demonstrating their role in impairing Ca²⁺-binding capacity and promoting protein misfolding [56, 79, 109]. These genetic defects reduce CASQ2’s ability to buffer Ca²⁺ ions in the SR, resulting in abnormal Ca²⁺ cycling, which is central to the pathophysiology of CPVT [110]. In individuals with CPVT, adrenergic stimulation, typically triggered by physical exertion or emotional stress [106] leads to excessive Ca²⁺ release from the SR. This abnormal release results from impaired CASQ2 function, causing DADs that can precipitate life-threatening arrhythmias, including bidirectional or polymorphic ventricular tachycardia [107, 111–113]. The compromised Ca²⁺ buffering capacity increases SR Ca²⁺ leak, further elevating cytosolic Ca²⁺ levels and heightening the risk of arrhythmogenic events.

### Risk of sudden cardiac death (SCD)

Untreated CPVT is highly lethal, with approximately 30% of affected individuals experiencing at least one cardiac arrest (CA), and up to 80% suffering recurrent syncope episodes [106]. Sudden death (SD) can be the initial manifestation, especially in children [114]. Patients presenting as the index case in a family (first diagnosed) generally have a more severe phenotype and are at a higher risk of adverse events, including SCD. Even after initiating medical treatment, high-risk ventricular arrhythmia persists in most patients, with fatal events occurring in 20.6% over 7.4 years, primarily due to non-compliance with exercise restrictions or medication [115]. Long-term follow-up studies show a high prevalence of cardiac events despite beta-blocker therapy, with 75% of patients experiencing syncope and 83% requiring implantable cardiac defibrillators [116]. More mutations and their associated diseases can be found in Table 1.

**Fig. 4:**
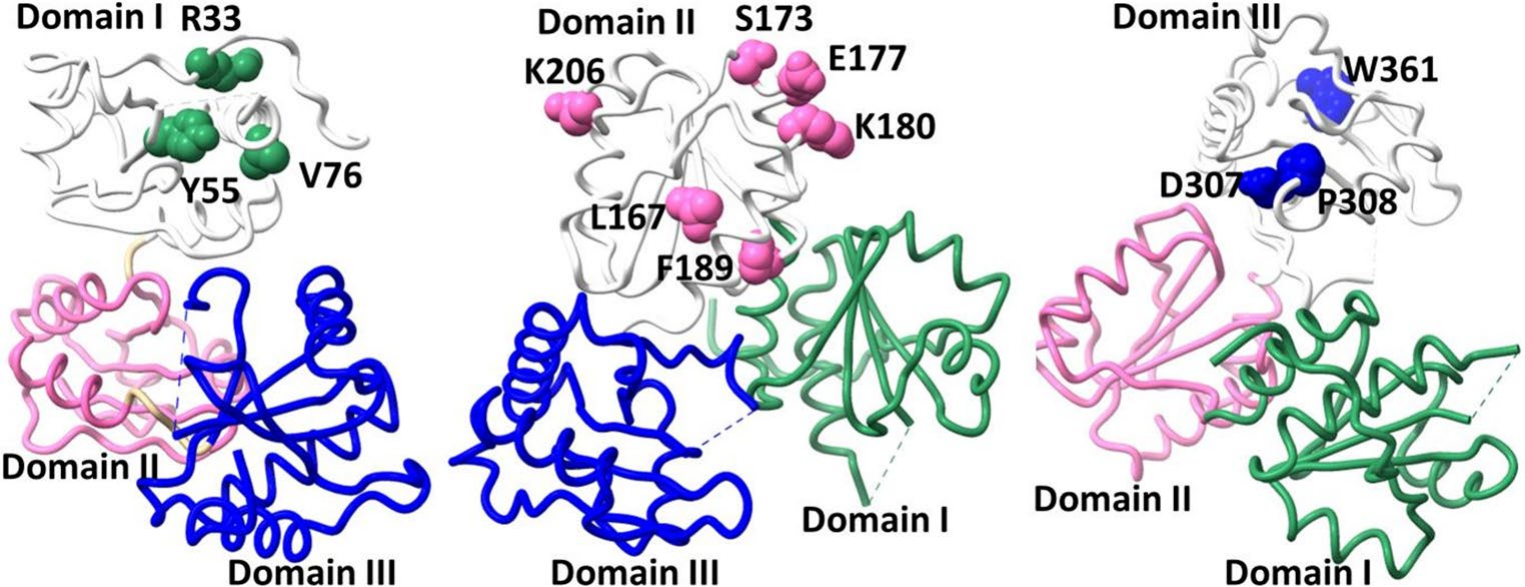
Location of disease associated mutations on the CASQ2 structure. CASQ2 monomeric crystal structure (PDBID: 6OWV) displaying the position of point mutations mentioned in Table 1. The locations of point mutations on CASQ2 domain I (left) are represented as spheres in green color. The locations of point mutations on CASQ2 domain II (middle) are shown as spheres in pink. Positions of domain III (right) mutations are shown as spheres in blue color. CASQ2 protein is colored domain-wise, domain I as green, domain II as pink and domain III as blue

Mutations in CASQ2 are highly lethal and several mutations disrupt filamentation [36]. Mapping of disease-causing point mutations onto the CASQ2 structure and its interfaces highlighted that the disease pathology is associated with defect in multimerization. The point mutations are distributed throughout the protein, and shared by three identically folded TrxL domains (domain I, domain II and domain III) (Fig. 4).

**Table 1:** Distinct CASQ2 mutations, pathologies they present in, and how they May lead to these pathologies

Most Common Associated Disease(s)/Phenotype(s)	Mutation: Amino Acid Change	Mutation: Nucleotide Change (cDNA Change)	Mutation: Type, Domain, Homozygosity	Proposed Underlying Mechanisms of Ca^2+^ Dysregulation/Functional Implication	Clinical Presentation/Notes/Severity
Catecholaminergic polymorphic ventricular tachycardia (CPVT)	D307H	1038 G > C [117, 118]	Missense, III, Homozygous while heterozygous carrier shows no symptoms [21, 119, 120]	Leads to either reduced SR Ca²⁺ content and impaired Ca²⁺ transients, or unaltered SR content with increased Ca²⁺ leak under both resting and stimulated conditions. CASQ2 expression is drastically reduced (~ 95%), and the mutant protein exhibits impaired Ca²⁺ buffering and binding, defective polymerization, and increased susceptibility to degradation. Although properly localized to the junctional SR, the mutant CASQ2 shows reduced interaction with triadin and junctin, and a loss of both low-affinity Ca²⁺ binding and Ca²⁺ selectivity—sometimes showing aggregation in the presence of Mg²⁺. Structural changes include protein misfolding and the loss of Ca²⁺-concentration-dependent conformational shifts. These alterations culminate in decreased SR Ca²⁺ storage capacity, impaired release, and the generation of spontaneous Ca²⁺ sparks [21, 42, 108, 117, 119–123]	Early onset during childhood and elevated mortality if left untreated, accompanied by resting bradycardia and a slight QTc interval prolongation [42, 108, 117, 119–123]
	R33Q	98 G > A [124]	Missense, N-terminus, Homozygous [21, 56, 125]	Exhibits ~ 50% reduced expression, impaired inhibition of RyR2, and increased RyR2 sensitivity to luminal Ca²⁺, leading to higher CICR gain, more frequent Ca²⁺ sparks and waves, and decreased SR Ca²⁺ content, despite unaltered triadin binding. Some variants show enhanced buffering, but with diminished Ca²⁺-induced conformational changes, reduced polymerization, and lower Ca²⁺ binding capacity at high concentrations, despite normal Ca²⁺ affinity. These defects are further exacerbated by increased protein degradation[21, 124–127]	Arrhythmia, non-sustained VT during exercise since young age which can lead to exercise-induced syncopal episodes [124]
	R33X	97 C > T [118]	Nonsense, N-terminus, Heterozygous [118, 128]	Introduces a premature stop codon, resulting in absence of full-length CASQ2 and a marked reduction in functional protein levels. This leads to disrupted Ca²⁺-dependent polymerization, with aggregation at low Ca²⁺ concentrations and impaired structural integrity. Despite correct localization to the junctional SR, the mutant protein fails to fully inhibit RyR2 at low luminal Ca²⁺, contributing to reduced SR Ca²⁺ content and the emergence of spontaneous Ca²⁺ waves and sparks [118, 128, 129]	Exercise-induced syncope, arrhythmias, and borderline QTc prolongation since childhood [118]
	L23fs + 14X	62delA [118]	Deletion, N-terminus, Homozygous while heterozygous siblings show no symptoms [118, 125]	The frameshift deletion causes a premature stop codon 14 amino acids downstream which results in truncated CASQ2 proteins that lack Ca²⁺-binding ability [118, 125]	Severe CPVT and exercise-induced syncope since young age [118]
	L167H	500 T > A [130]	Missense, II, Compound heterozygous with **G112 + 5X** [42, 108, 130]	Increases sensitivity to proteolytic cleavage and reduces Ca²⁺-dependent polymerization. While Ca²⁺-binding affinity is preserved, binding capacity declines at high Ca²⁺ levels, and the protein aggregates at low Ca²⁺. It also loses Ca²⁺-induced conformational changes, impairing RyR2 activation at 1 mM Ca²⁺. These defects lead to reduced SR Ca²⁺ content, diminished SR Ca²⁺ release, and fewer spontaneous Ca²⁺ sparks [21, 42, 108, 129, 130]	Severe CPVT with several runs of polymorphic ventricular tachycardia and multiple syncopal events from young age [130]
	Y55C	164 A > G [131]	Missense, I, Compound heterozygous with **P308L** [125, 131]	Loss of proper dimerization and filamentation capabilities [125, 131]	Non-sustained VT under adrenaline stress; requires compound heterozygosity with P308L to manifest full CPVT phenotype [131]
	V76M	226G > A [132]	Missense SNP, I, Heterozygous [132, 133]	Disrupts protein stability and polymerization, leading to reduced expression levels. It also partially impairs Ca²⁺ binding capacity [132, 133]	Shows no consistent clinical presentation and is considered a rare, likely benign polymorphism found in Finnish families & Asian population [132, 133]
Sudden Death (SD)	E177Q	529G > C [132]	Missense, II, Heterozygous mutation [132]	Suggested disruption of Ca²⁺ binding capacity due to substitution of a negatively charged glutamic acid with a neutral glutamine which may impair normal chelation function. However, no experimental functional studies were performed [132]	No clinical symptoms reported; sudden unexplained death [132]
	S173I	518G > T [134]	Missense, II (at the inter-dimer interface with III), Heterozygous [36]	Disrupts CASQ2 filamentation by interfering with Ca²⁺-induced polymer formation at the inter-dimer interface, leading to defective Ca²⁺ buffering in the SR [36]	CPVT-like and sudden unexplained death at young age [36]
CPVT, Cardiac Arrest (CA)	G112 + 5X	339del16 [130]	Deletion, II, III, and part of I, Homozygous [130]	A 16 bp frameshift deletion in exon 3 introduces a premature stop codon, resulting in a truncated CASQ2 protein lacking ~ 260 downstream amino acids; this mutant cannot bind Ca²⁺ (owing to the loss of most of the acidic residues at the C-terminal), lacks key domains required for front-to-front and back-to-back interactions, and leads to decreased SR Ca²⁺ storage, reduced SR Ca²⁺ release, and increased spontaneous Ca²⁺ sparks [130]	Exhibiting a severe form of CPVT, characterized by CA, exercise-induced syncope, rapid polymorphic ventricular tachycardia, and stress-triggered VT starting from young age [130]
	K206N	618 A > C [135]	Missense, II, Heterozygous, Heterozygous carrier [135]	The mutation introduces an extra N-glycosylation site, leading to reduced Ca²⁺-binding capacity and impaired oligomer formation. This disrupts CASQ2 polymerization, enhances its interaction with RyR2, and increases the open probability of RyR2 Ca²⁺ channels. As a result, Ca²⁺ leak is elevated under both basal and stimulated conditions, SR Ca²⁺ concentration is reduced, and spontaneous Ca²⁺ release events in cardiomyocytes are more frequent. Despite these changes, CASQ2’s association with triadin remains unaffected [135]	Syncope induced by emotion, stress, swimming and exercise since young age along with bradycardia and multiple extrasystoles despite normal QTc interval [135]
	W361X	1083G > A [136]	Nonsense, C-terminus, Homozygous [136]	Causes premature truncation of the protein’s C-terminal domain, impairing its Ca²⁺-buffering function in the SR. This leads to excess free Ca²⁺, resulting in diastolic Ca²⁺ leak, which triggers arrhythmias under stress, manifesting as CPVT [136]	Syncopal episodes since childhood, triggered by exercise and emotional stress. Initially misdiagnosed with epilepsy; ECG later showed left axis deviation and QT-U prolongation. Exercise testing induced polymorphic ventricular ectopy developing into polymorphic VT with syncope [136]
CPVT, SD	F189L	567 C > G [137]	Missense, II, Heterozygous [137, 138]	Potential decrease in protein mobility [137–139]	SD while exertion in the context of underlying, early-onset CPVT
	P308L	923 C > T [131]	Missense, III, Compound heterozygous [42, 131]	Maintains dimerization yet fails to properly polymerize and interact with RyR2; causes conformational changes; Ca²⁺ selectivity is lost, and Mg2 + triggers aberrant aggregation [42, 125, 131, 140]	Early-onset, exercise-induced syncope progressing to severe CPVT [131]
CPVT, CA, SD	-	532 + 1G > A [118]	Incorrect splicing, Homozygous intronic base pair change, While homozygous carrier individuals show symptoms, heterozygous siblings remain unaffected, indicating recessive inheritance [118, 128, 141]	Leads to skipping exon 4, which introduces a frameshift and premature stop codon, preventing the production of full-length CASQ2 and resulting in a total loss of functional CASQ2 protein [118, 128, 141]	Causes a severe form of CPVT in homozygous individuals, presenting in early childhood with syncope or CA triggered by exercise [118]
	K180R	539 A > G [142]	Missense, II, Heterozygous dominant inheritance [36, 142]	The mutation maintains dimerization but disrupts inter-dimer interactions, impairing polymerization and resulting in a defective filament structure that alters interaction with IRE1α, while also impairing dynamic Ca²⁺ buffering in the SR without affecting total CASQ2 protein levels or overall SR Ca²⁺ content [36, 125, 142]	It causes severe CPVT with early-onset symptoms. Clinical findings revealed exercise-induced ventricular arrhythmia and syncope [142]

CA – Cardiac Arrest; Ca²⁺ – Calcium ion; CASQ2 – Calsequestrin 2; CICR – Calcium-Induced Calcium Release; CPVT – Catecholaminergic Polymorphic Ventricular Tachycardia; DADs – Delayed Afterdepolarizations; ECG – Electrocardiogram; IRE1α – Inositol-Requiring Enzyme 1 alpha; Mg²⁺ – Magnesium ion; QTc – Corrected QT Interval; RyR2 – Ryanodine Receptor 2; SD – Sudden Death; SR – Sarcoplasmic Reticulum; VT – Ventricular Tachycardia

### Age and disease severity

The severity of CPVT exhibits marked age-dependent heterogeneity, with pediatric patients facing substantially higher risks of life-threatening arrhythmias during physical or emotional stress. The disease burden is particularly pronounced in children and adolescents, who demonstrate a 4-fold higher incidence of cardiac events compared to adults, with approximately one-third of patients experiencing their first symptomatic event before age 21 [143, 144]. These trends are primarily derived from studies of CPVT1, which represents the majority of reported cases.

In contrast, CPVT2 data remain limited, though available reports suggest a relatively later onset and milder arrhythmic phenotype in many patients. This apparent difference may reflect distinct molecular mechanisms, as RyR2 mutations directly alter the release channel, whereas CASQ2 mutations primarily affect luminal Ca²⁺ buffering.

Adult-onset CPVT cases, including those due to CASQ2 mutations, often show attenuated phenotypic expression with extended asymptomatic periods; however, they remain at risk of sudden cardiac death without warning. Notably, the 40-year arrhythmia-free survival rate for asymptomatic RyR2 mutation carriers is only 27%, underscoring the persistent risk across all age groups despite the apparent clinical quiescence in some adults [143], underscoring that age does not fully mitigate risk across CPVT subtypes. While extrapolation from CPVT1 to CPVT2 should be made with caution, both disorders share the central feature of stress-induced Ca²⁺ dysregulation as a driver of arrhythmic vulnerability.

### Arrhythmias

Beyond CPVT, CASQ2 mutations are implicated in a broader spectrum of arrhythmias, including AF and sinus node dysfunction (SND) [74, 145, 146].

Normal cardiac automaticity depends on the cardiac sinoatrial node’s (SAN) pacemaker cells. Human SND is caused by cardiac pace-making disorder. SND is more common in older adults and is linked to irregular cardiac rhythms caused by compromised pace-maker function [147].

Sinoatrial node block, bradycardia/tachycardia syndrome, syncope, sinus bradycardia, and sinus arrest are among the symptoms that people with SND experience. Crucially, whether under stress or exercising, people with SND report experiencing chronotropic incompetence. SND can be inherited or linked to cardiovascular or systemic diseases. The only treatments available to patients with SND at this time are pace-maker implantation when necessary and the alleviation of arrhythmia symptoms [147].

CASQ2-deficient mouse models exhibit atrial ectopy, conduction block, and enhanced susceptibility to AF under adrenergic stimulation. As one study showed CPVT patients have both SAN and atrioventricular node dysfunction, and that these conditions are frequently linked to either spontaneous or exercise/catecholomine-induced AF. This suggests that the supraventricular tissues and the ventricle are involved in the disease’s background arrhythmogenic substrate as opposed to just the ventricular tissue as previously hypothesized. This could potentially be an indication for close follow-up with an implantable cardioverter-defibrillator (ICD) to monitor for AF [145]. Furthermore, structural remodeling, such as fibrosis in the sinoatrial node region, disrupts pacemaker activity and predisposes to SND [56].

## Therapeutic approach

Efforts to restore CASQ2 functionality have led to diverse therapeutic strategies, including pharmacological interventions, molecular therapies, and emerging gene-based approaches aimed at improving Ca²⁺ homeostasis and reducing arrhythmogenic events.

### Current interventions

#### Pharmacological

Reducing the frequency of arrhythmias is the primary objective of treatment. This can be accomplished by combining many strategies, including left cardiac sympathetic denervation, implantable cardiac defibrillators, medication treatments, and lifestyle modifications. Because lifestyle activities such as stressful circumstances, demanding workloads, and competitive sports lead to the release of catecholamines, which in turn can induce CPVT. As a result, guidelines suggest avoiding such activities.

Beta-blockers remain the first-line treatment for CASQ2-related arrhythmias [148]. These drugs mitigate adrenergic stimulation, which reduces intracellular Ca²⁺ overload and the risk of triggered arrhythmias. Propranolol and nadolol are widely prescribed [149, 150], but many patients experience breakthrough arrhythmias, necessitating adjunctive treatments. Flecainide, a sodium channel blocker, has shown efficacy in suppressing arrhythmias; however, its mechanisms of action remain unclear, with evidence pointing towards effects on both RyR2 and sodium channels, and to a minor extent on sodium-calcium exchanger, which collectively reduce DADs [151]. Despite their benefits, these pharmacological options often fail to provide comprehensive arrhythmia control [149, 152], highlighting the need for targeted therapies specifically addressing CASQ2 dysfunction.

Recent experimental evidence highlights melatonin as a promising adjunct in managing arrhythmias linked to impaired Ca²⁺ handling, particularly in heart failure settings. In acute ischemia-reperfusion (IR)-injured rabbit models, melatonin administration significantly reduced ventricular fibrillation maintenance and suppressed spatially discordant alternans - electrophysiological precursors of life-threatening arrhythmias [153, 154]. This antiarrhythmic effect was associated with enhanced SR Ca²⁺ handling, partly through upregulation of CASQ2 and SERCA2a expression, along with attenuation of phosphorylated phospholamban downregulation in failing hearts [153]. Notably, melatonin’s beneficial effects on Ca²⁺ homeostasis were partially counteracted by therapeutic hypothermia, underscoring the importance of temperature-sensitive mechanisms in its efficacy.

However, these findings were derived from heart-failure and ischemic models rather than CASQ2-mutant systems. In CPVT2, most mutations are homozygous and result in structurally defective CASQ2 proteins, meaning that simply increasing the amount of mutated protein may not restore normal RyR2 interactions or Ca^2+^ buffering. Therefore, the direct benefit of melatonin-induced CASQ2 upregulation in CPVT2 remains uncertain and should be interpreted cautiously.

While not traditionally classified as an antiarrhythmic drug, melatonin’s ability to improve SR Ca²⁺ storage capacity via CASQ2 modulation in experimental settings suggests potential utility in conditions marked by defective Ca²⁺ buffering and diastolic Ca²⁺ leak. In CPVT2, however, greater SR Ca²⁺ loading through SERCA2a upregulation could theoretically worsen RyR2 hyperactivity, so further research is needed to understand this effect. Its antioxidant and anti-inflammatory properties further support cardioprotection in IR injury models, positioning melatonin as a multifunctional agent that may complement existing pharmacotherapies for arrhythmias related to impaired Ca²⁺ handling, although its role in CASQ2-related CPVT2 remains speculative at present [155].

#### Non-pharmacological

Patients with CPVT require comprehensive lifestyle and medical management to mitigate arrhythmia risks. Current guidelines strongly advise against competitive sports and high-intensity physical activities such as sprinting or basketball due to the well-documented risk of adrenergically-triggered ventricular arrhythmias. However, carefully supervised mild-to-moderate aerobic exercise like walking or light cycling may be permissible for select patients who demonstrate excellent arrhythmia control on medication and have no history of cardiac events [156]. Regular follow-up with a cardiologist specializing in inherited arrhythmias is crucial, particularly during high-risk periods like puberty, pregnancy, and growth spurts when hormonal changes may increase arrhythmia susceptibility.

For patients with refractory arrhythmias or multiple ICD shocks despite optimal pharmacotherapy, left cardiac sympathetic denervation (LCSD) represents an important intervention [157]. This surgical procedure involves the resection of the lower half of the left stellate ganglion and the first thoracic ganglia (T1-T4), significantly reducing norepinephrine release to the heart. While the majority of clinical evidence comes from patients with CPVT1, LCSD is also considered for high-risk CPVT2 patients, effectively reducing the burden of life-threatening arrhythmias by blunting the sympathetic trigger [158].

Genetic counseling forms an essential component of CPVT care, providing family risk assessment through cascade genetic testing and discussing reproductive options including prenatal diagnosis and preimplantation genetic testing. Comprehensive diagnostic evaluation combines exercise stress testing, which provokes diagnostic ventricular arrhythmias in approximately 75% of cases, with molecular genetic testing that identifies pathogenic variants in 60–70% of clinically diagnosed patients [106]. Emerging therapeutic options including flecainide for RYR2-mediated CPVT and novel Rycal compounds show promise in clinical trials, though beta-blockers remain the mainstay of therapy. All patients require individualized management plans incorporating these elements to optimize outcomes while maintaining quality of life [106, 156].

### Emerging therapies and future directions

#### CASQ2 stabilizers

Emerging pharmacological treatments for CPVT aim to correct the pathways responsible for arrhythmogenesis. Research has highlighted the effectiveness of K201, a 1,4-benzothiazepine derivative, in stabilizing calstabin2-RyR2 binding, likely due to its structural similarity to diltiazem [159, 160]. This stabilization has been shown to prevent arrhythmias.

Another innovative agent, ent-(+)-verticilide, a synthetic derivative of the insecticide verticilide, was found to reduce delayed after-depolarizations by selectively inhibiting RyR2 Ca²⁺ release in murine cardiac cells with CASQ2 mutations. While its exact mechanism of action remains unclear, it appears to act independently of RyR2 phosphorylation or interactions with accessory proteins [161]. Conversely, both dantrolene and K201 target two of the three known mechanisms of RyR2 dysfunction. Dantrolene has shown success in reducing arrhythmias in patients with RyR2 domain defects, while clinical trials for K201 and ent-(+)-verticilide have yet to be conducted [162].

#### Gene therapy and stem cell models

Gene therapy has emerged as a revolutionary approach for addressing CASQ2-related pathologies, with adeno-associated virus (AAV) vectors becoming the leading delivery platform due to their cardiac tropism and long-term transgene expression. Preclinical studies using AAV9-CASQ2 in CPVT mouse models demonstrated > 80% restoration of CASQ2 protein levels, resulting in complete suppression of ventricular arrhythmias during adrenergic challenge and improved 6-month survival rates from 40% to 90% [163–169]. Notably, AAV-mediated CASQ2 expression was shown to functionally compensate for both loss-of-function mutations and CASQ2 knockout phenotypes by normalizing SR Ca^2+^ buffering capacity [163–169].

Complementary to gene therapy, human-induced pluripotent stem cell (hiPSC) models from CPVT patients have enabled unprecedented mechanistic studies of CASQ2 dysfunction. These patient-specific cardiomyocyte models recapitulate key disease features including abnormal Ca^2+^ transients and delayed afterdepolarizations, serving as platforms for drug screening [170]. Recent hiPSC studies identified flecainide and carvedilol as particularly effective in CASQ2-deficient cells, with combination therapy showing synergistic benefits [171, 172].

Recent advances in iPSC modeling have enabled precise investigation of CASQ2-related pathophysiology. A new isogenic Clustered Regularly Interspaced Short Palindromic Repeats (CRISPR)/Cas9-corrected iPSC line derived from a CPVT patient carrying a heterozygous mutation in CASQ2 has been established, offering a powerful platform to dissect molecular mechanisms and evaluate therapeutic interventions [173, 174]. These gene-edited iPSCs were shown to retain normal karyotype, pluripotency markers, and absence of off-target mutations, making them suitable for differentiating into cardiomyocytes that recapitulate patient-specific Ca²⁺ handling abnormalities [175]. Such models allow for high-fidelity comparisons between mutant and corrected lines, supporting the development of precision therapies targeting CASQ2 dysfunction and associated arrhythmias.

Parallel therapeutic development includes small molecule modulators targeting the CASQ2-RyR2 interaction, such as the Rycal compound S107 which stabilizes the RyR2-calstabin2 complex and reduces diastolic Ca^2+^ leak in CASQ2-mutant cardiomyocytes [176, 177]. Second-generation derivatives with improved bioavailability are currently in Phase I/II trials for CPVT. These approaches collectively address the fundamental pathophysiology of CASQ2 deficiency: impaired Ca^2+^ buffering, aberrant RyR2 regulation, and consequent pro-arrhythmic Ca^2+^ waves.

### Challenges in developing CASQ2-Targeted therapies

The development of CASQ2-targeted therapies faces several challenges. Genetic heterogeneity among CASQ2 mutations complicates the creation of universal treatments, requiring personalized approaches [178, 179]. Delivering therapies specifically to cardiac tissue without affecting skeletal muscle isoforms presents additional obstacles [163, 180]. Moreover, the long-term safety and efficacy of emerging therapies, including gene-based interventions, remain critical areas of concern. Addressing these challenges will be essential for translating preclinical successes into viable clinical treatments [114].

Therapeutic approaches targeting CASQ2 have advanced significantly, encompassing pharmacological, molecular, and gene-based strategies. While current interventions offer partial symptomatic relief and reduced risk of adverse events, emerging therapies hold the promise of addressing the underlying molecular dysfunctions of CASQ2. Overcoming challenges related to genetic variability, delivery specificity, and long-term safety will be pivotal in realizing the full potential of these innovative treatments and improving outcomes for patients with CASQ2-related arrhythmias [114, 163, 180].

These strategies are summarized in Table 2, which outlines current, emerging, and experimental approaches for targeting CASQ2-related dysfunction.

**Table 2:** Therapeutic strategies for CASQ2-Related catecholaminergic polymorphic ventricular tachycardia (CPVT)

Category	Therapeutic Strategy	Mechanism of Action	Key Insights
Current Interventions	**Beta-blockers (e.g.**,** propranolol**,** nadolol)**	Block β-adrenergic stimulation to reduce intracellular Ca²⁺ overload	First-line treatment for CPVT; reduce risk of triggered arrhythmias but may not fully suppress symptoms in all patients
	**Flecainide**	Blocks sodium channels and inhibits spontaneous Ca²⁺ release through RyR2	Reduces delayed afterdepolarizations (DADs); often used in combination with beta-blockers for better control
	**Lifestyle Modifications**	Avoid physical/emotional stress that triggers adrenergic surges	Critical for preventing arrhythmic episodes; high-intensity sports discouraged, light activity allowed under supervision
	**Implantable Cardioverter-Defibrillator (ICD)**	Provides immediate correction of life-threatening arrhythmias	Considered for high-risk or drug-refractory cases
	**Left Cardiac Sympathetic Denervation (LCSD)**	Surgical resection of left stellate/T1-T4 ganglia to reduce cardiac norepinephrine release	Reserved for high-risk, drug-refractory patients; reduces arrhythmia burden and ICD shocks by blunting sympathetic trigger.
	**Genetic Counseling & Testing**	Identifies carriers and guides reproductive planning	Enables early diagnosis in family members; supports personalized care strategies
Emerging Therapies	**K201 (JTV519)**	Enhances RyR2-calstabin2 binding, stabilizing RyR2 channel	Prevents pathological Ca²⁺ leak; promising in preclinical models; related structurally to diltiazem
	**Dantrolene**	Restores RyR2 interdomain stability and normalizes gating	Shown to reduce ventricular arrhythmias in mutant RyR2 models; targets defective channel conformation
	**ent-(+)-Verticilide**	Selectively inhibits RyR2-mediated Ca²⁺ release	Reduces DADs in CASQ2-mutant cells; mechanism independent of RyR2 phosphorylation or accessory proteins
	**S107 (Rycal compound)**	Stabilizes RyR2–calstabin2 complex to reduce Ca²⁺ leak	Reduces diastolic Ca²⁺ leak in CASQ2-deficient cardiomyocytes; second-generation derivatives in early clinical trials
	**Melatonin**	Enhances SR Ca²⁺ handling through upregulation of CASQ2 and SERCA2a; modulates phospholamban phosphorylation	Demonstrated to reduce ventricular fibrillation and Ca²⁺ alternans in IR injury models; also offers antioxidant and anti-inflammatory cardioprotection. Evidence derived from heart-failure models; potential relevance to CASQ2-related CPVT2 remains speculative.
Gene Therapy	**AAV9-CASQ2 Gene Transfer**	Replaces defective CASQ2 gene using cardiac-targeted AAV9 vector	Demonstrated > 80% restoration of CASQ2 expression and suppression of arrhythmias in CPVT mouse models; improved survival rates
Patient-Specific Models	**hiPSC-derived Cardiomyocytes**	Replicate patient-specific CASQ2 defects for drug screening	Reveal abnormal Ca²⁺ handling and arrhythmogenic behavior; enable evaluation of personalized drug responses (e.g., flecainide + carvedilol synergy)
	**CRISPR/Cas9-Corrected iPSC Model**	Isogenic mutation correction in patient-derived iPSCs	Provides high-fidelity platform for comparing mutant vs. corrected lines; supports discovery of targeted therapies for CASQ2-related arrhythmias
Challenges	**Genetic Heterogeneity**	Wide range of CASQ2 mutations leads to variable functional impact	Limits the development of universal therapies; personalized strategies are needed
	**Tissue-Specific Targeting**	Avoiding off-target effects in non-cardiac tissues (e.g., skeletal muscle)	Critical for gene therapies and small molecules with systemic delivery
	**Long-Term Safety & Efficacy**	Especially relevant for gene-based and novel molecular interventions	Requires careful long-term follow-up and monitoring of therapeutic durability and side effects

AAV – Adeno-associated virus; AAV9 – Adeno-associated virus serotype 9; β – Beta; Ca²⁺ – Calcium ion; CASQ2 – Calsequestrin 2; CPVT – Catecholaminergic Polymorphic Ventricular Tachycardia; DADs – Delayed Afterdepolarizations; ICD – Implantable Cardioverter-Defibrillator; IR – Ischemia-Reperfusion; LCSD – Left Cardiac Sympathetic Denervation; RyR2 – Ryanodine Receptor 2; SERCA2a – Sarcoplasmic Reticulum Ca²⁺-ATPase 2a; SR – Sarcoplasmic Reticulum; hiPSC – human-induced Pluripotent Stem Cell; CRISPR – Clustered Regularly Interspaced Short Palindromic Repeats; iPSC – induced Pluripotent Stem Cell; CASQ – Calsequestrin; VT – Ventricular Tachycardia

## Conclusion

Calsequestrin 2 is a key protein in cardiac muscle physiology, serving as the primary Ca²⁺-binding protein in the SR. Its role extends beyond Ca²⁺ buffering, as it actively regulates Ca²⁺ release through interactions with RyRs, triadin, and junctin. These interactions ensure precise Ca²⁺ handling, which is fundamental for maintaining cardiac rhythm and contraction efficiency. The structural organization of CASQ2, including its thioredoxin-like domains and intrinsically disordered C-terminal region, allows it to dynamically respond to fluctuations in luminal Ca²⁺ concentrations, ensuring proper EC coupling.

Dysfunction in CASQ2 leads to severe cardiac pathologies, most notably CPVT, an inherited arrhythmogenic disorder that significantly increases the risk of SCD. Mutations in the CASQ2 gene disrupt its Ca²⁺-binding capacity and polymerization dynamics, leading to spontaneous Ca²⁺ release, delayed afterdepolarizations, and ventricular arrhythmias. Despite advances in pharmacological treatments such as beta-blockers and flecainide, many patients remain at high risk, highlighting the urgent need for more targeted therapies.

Emerging therapeutic strategies, including CASQ2 stabilizers, gene therapy, and small-molecule modulators, offer promising avenues for restoring normal Ca²⁺ homeostasis. While challenges remain, such as genetic heterogeneity and tissue-specific targeting, ongoing research is paving the way for precision medicine approaches in CASQ2-related arrhythmias. Future advancements in molecular therapies and gene-editing techniques may ultimately provide curative treatments, transforming the management of CASQ2-associated cardiac diseases. Through continued exploration, CASQ2 remains not only a fundamental component of Ca²⁺ regulation but also a key target in the quest to mitigate arrhythmogenic cardiac disorders.

## Data Availability

No datasets were generated or analysed during the current study.

## Abbreviations

AAV: Adeno–associated virus
AF: Atrial Fibrillation
ARVC: Arrhythmogenic Right Ventricular Cardiomyopathy
Ca²⁺: Calcium ion
CArG: CC(A/T)₆GG box
CASQ: Calsequestrin
CASQ1: Calsequestrin 1 (skeletal muscle isoform)
CASQ2: Calsequestrin 2 (cardiac muscle isoform)
CA: Cardiac Arrest
CICR: Calcium–induced Calcium Release
CPVT: Catecholaminergic Polymorphic Ventricular Tachycardia
CRISPR: Clustered Regularly Interspaced Short Palindromic Repeats
DADs: Delayed Afterdepolarizations
EADs: Early Afterdepolarizations
EC: Excitation–Contraction
ECG: Electrocardiogram
ER: Endoplasmic Reticulum
hiPSC: human–induced Pluripotent Stem Cell
ICD: Implantable Cardioverter–Defibrillator
IR: Ischemia–Reperfusion
IRE1α: Inositol–Requiring Enzyme 1 alpha
jSR: Junctional Sarcoplasmic Reticulum
KEKE: Lysine (K)–Glutamate (E)–Lysine (K)–Glutamate (E) motif
MEF: 2–Myocyte Enhancer Factor–2
MEF2C: Myocyte Enhancer Factor 2 C
NFAT: Nuclear Factor of Activated T–cells
PDI: Protein Disulfide Isomerase
QTc: Corrected QT Interval
RyR: Ryanodine Receptor
RyR2: Ryanodine Receptor 2 (cardiac isoform)
SAN: Sinoatrial Node
SCD: Sudden Cardiac Death
SD: Sudden Death
SERCA: Sarcoplasmic Reticulum Ca²⁺–ATPase
SOCE: Store–Operated Calcium Entry
SND: Sinus Node Dysfunction
SNP: Single Nucleotide Polymorphism
SR: Sarcoplasmic Reticulum
SRF: Serum Response Factor
SRJ: Sarcoplasmic Reticulum Junction
STIM1: Stromal Interaction Molecule 1
TrxL: Thioredoxin–like
UPR: Unfolded Protein Response
VT: Ventricular Tachycardia

## References

[1] Pu F, Chen N, Xue S (2016) Calcium intake, calcium homeostasis and health. Food Sci Hum Wellness 5(1):8–16

[2] Calcium R, Weekly (2021) 1885(1):124–124

[3] Rajagopal S, Ponnusamy M (2017) Regulation of Calcium in Muscle Physiology. In: Rajagopal S, Ponnusamy M (eds) Calcium Signaling: From Physiology to Diseases. Springer Singapore, Singapore, pp 15–30

[4] Eisner DA et al (2017) Calcium and Excitation-Contraction coupling in the heart. Circ Res 121(2):181–195 28684623 10.1161/CIRCRESAHA.117.310230 PMC5497788

[5] Aronsen JM, Louch WE, Sjaastad I (2016) Cardiomyocyte Ca2 + dynamics: clinical perspectives. Scand Cardiovasc J 50(2):65–77 26729487 10.3109/14017431.2015.1136079

[6] Serano M et al (2025) Intracellular membrane contact sites in skeletal muscle cells. Membranes 15(1):29 39852269 10.3390/membranes15010029 PMC11767089

[7] Rossi D et al (2021) Calsequestrin, a key protein in striated muscle health and disease. J Muscle Res Cell Motil 42(2):267–279 32488451 10.1007/s10974-020-09583-6

[8] Woo JS et al (2020) Calsequestrin: a well-known but curious protein in skeletal muscle. Exp Mol Med 52(12):1908–1925 33288873 10.1038/s12276-020-00535-1 PMC8080761

[9] MacLennan D.H., Wong P.T.S. (1971) Isolation of a Calcium-Sequestering protein from sarcoplasmic reticulum. Proc Natl Acad Sci 68(6):1231–1235 4256614 10.1073/pnas.68.6.1231 PMC389160

[10] Faggioni M, Knollmann BC (2012) Calsequestrin 2 and arrhythmias. Am J Physiol Heart Circ Physiol 302(6):H1250–H1260 22198169 10.1152/ajpheart.00779.2011 PMC3311477

[11] Handhle A et al (2016) Calsequestrin interacts directly with the cardiac Ryanodine receptor luminal domain. J Cell Sci 129(21):3983–3988 27609834 10.1242/jcs.191643 PMC5117208

[12] Sibbles ET et al (2022) The function and regulation of calsequestrin-2: implications in calcium-mediated arrhythmias. Biophys Rev 14(1):329–352 35340602 10.1007/s12551-021-00914-6 PMC8921388

[13] Wang S et al (1998) Crystal structure of calsequestrin from rabbit skeletal muscle sarcoplasmic reticulum. Nat Struct Biol 5(6):476–483 9628486 10.1038/nsb0698-476

[14] Wleklinski MJ et al (2022) Impaired dynamic sarcoplasmic reticulum Ca buffering in autosomal dominant CPVT2. Circ Res 131(8):673–686 36102198 10.1161/CIRCRESAHA.121.320661 PMC9529867

[15] Boncompagni S et al (2012) Triadin/Junctin double null mouse reveals a differential role for Triadin and junctin in anchoring CASQ to the jSR and regulating Ca2 + Homeostasis. PLoS ONE 7(7):e39962 22768324 10.1371/journal.pone.0039962 PMC3388061

[16] Lee KW et al (2012) Role of junctin protein interactions in cellular dynamics of calsequestrin polymer upon calcium Perturbation *. J Biol Chem 287(3):1679–1687 22123818 10.1074/jbc.M111.254045 PMC3265851

[17] Dulhunty AF et al (2017) Core skeletal muscle Ryanodine receptor calcium release complex. Clin Exp Pharmacol Physiol 44(1):3–12 27696487 10.1111/1440-1681.12676

[18] Otsu K et al (1993) Chromosome mapping of five human cardiac and skeletal muscle sarcoplasmic reticulum protein genes. Genomics 17(2):507–509 8406504 10.1006/geno.1993.1357

[19] Scott BT et al (1988) Complete amino acid sequence of canine cardiac calsequestrin deduced by cDNA cloning. J Biol Chem 263(18):8958–8964 3379055

[20] Fliegel L et al (1987) Amino acid sequence of rabbit fast-twitch skeletal muscle calsequestrin deduced from cDNA and peptide sequencing. Proc Natl Acad Sci 84(5):1167–1171 3469659 10.1073/pnas.84.5.1167 PMC304387

[21] Kim E et al (2007) Characterization of human cardiac calsequestrin and its deleterious mutants. J Mol Biol 373(4):1047–1057 17881003 10.1016/j.jmb.2007.08.055

[22] Hornbeck PV et al (2015) PhosphoSitePlus, 2014: mutations, PTMs and recalibrations. Nucleic Acids Res 43(D1):D512–D520 25514926 10.1093/nar/gku1267 PMC4383998

[23] Lewis KM et al (2016) Characterization of Post-Translational modifications to calsequestrins of cardiac and skeletal muscle. Int J Mol Sci 17(9):1539 27649144 10.3390/ijms17091539 PMC5037814

[24] Park H et al (2003) Polymerization of calsequestrin. Implications for Ca2 + regulation. J Biol Chem 278(18):16176–16182 12594204 10.1074/jbc.M300120200

[25] Brodersen DE, Kjeldgaard M (1999) Structural investigations of calcium and zinc binding in proteins. Sci Prog 82(4):295–312 10701337 10.1177/003685049908200402 PMC10367491

[26] Palma JH, Bertuola M, Hermida ÉB (2024) Modeling calcium diffusion and crosslinking dynamics in a thermogelling Alginate-Gelatin-Hyaluronic acid ink: 3D Bioprinting applications. Bioprinting 38:e00329

[27] Stewart WW, Swaisgood HE (1993) Characterization of calcium alginate pore diameter by size-exclusion chromatography using protein standards. Enzym Microb Technol 15(11):922–927

[28] Santamaria-Kisiel L, Rintala-Dempsey AC, Shaw GS (2006) Calcium-dependent and -independent interactions of the S100 protein family. Biochem J 396(2):201–214 16683912 10.1042/BJ20060195 PMC1462724

[29] Yang M et al (2006) Calcium-dependent dimerization of human soluble calcium activated nucleotidase: Characterization of the dimer interface*. J Biol Chem 281(38):28307–28317 16835225 10.1074/jbc.M604413200

[30] Galligan JJ, Petersen DR (2012) The human protein disulfide isomerase gene family. Hum Genomics 6:1–15 23245351 10.1186/1479-7364-6-6 PMC3500226

[31] Atkinson HJ, Babbitt PC (2009) An atlas of the thioredoxin fold class reveals the complexity of Function-Enabling adaptations. PLoS Comput Biol 5(10):e1000541 19851441 10.1371/journal.pcbi.1000541 PMC2757866

[32] Banci L et al (2007) A structural characterization of human SCO2. Structure 15(9):1132–1140 17850752 10.1016/j.str.2007.07.011

[33] Park H et al (2004) Comparing skeletal and cardiac calsequestrin structures and their calcium binding: A PROPOSED MECHANISM FOR COUPLED CALCIUM BINDING AND PROTEIN POLYMERIZATION *. J Biol Chem 279(17):18026–18033 14871888 10.1074/jbc.M311553200

[34] Beard NA, Dulhunty AF (2015) C-terminal residues of skeletal muscle calsequestrin are essential for calcium binding and for skeletal ryanodine receptor inhibition. Skelet Muscle. 10.1186/s13395-015-0029-7 25861445 10.1186/s13395-015-0029-7 PMC4389316

[35] Kang C, Trumble WR, Dunker AK (2002) Crystallization and Structure-Function of calsequestrin. Calcium-Binding protein protocols: volume 1: reviews and case studies. Humana, Totowa, NJ, pp 281–294. H.J. Vogel, Editor 10.1385/1-59259-183-3:281 11833354

[36] Titus EW et al (2020) The structure of a calsequestrin filament reveals mechanisms of Familial arrhythmia. Nat Struct Mol Biol 27(12):1142–1151 33046906 10.1038/s41594-020-0510-9 PMC7718342

[37] Aaron BM et al (1984) Characterization of skeletal muscle calsequestrin by 1H NMR spectroscopy. J Biol Chem 259(19):11876–11881 6480588

[38] Donoso P, Beltrán M, Hidalgo C (1996) Luminal pH regulates calcium release kinetics in sarcoplasmic reticulum vesicles. Biochemistry 35(41):13419–13425 8873610 10.1021/bi9616209

[39] Hidalgo C, Donoso P, Rodriguez PH (1996) Protons induce calsequestrin conformational changes. Biophys J 71(4):2130–2137 8889188 10.1016/S0006-3495(96)79413-4 PMC1233680

[40] Krause KH et al (1991) Thermodynamics of cation binding to rabbit skeletal muscle calsequestrin. Evidence for distinct Ca(2+)- and Mg(2+)-binding sites. J Biol Chem 266(15):9453–9459 2033046

[41] Ostwald TJ, MacLennan DH, Dorrington KJ (1974) Effects of cation binding on the conformation of calsequestrin and the high affinity calcium-binding protein of sarcoplasmic reticulum. J Biol Chem 249(18):5867–5871 4472093

[42] Bal NC et al (2011) Probing cationic selectivity of cardiac calsequestrin and its CPVT mutants. Biochem J 435(2):391–399 21265816 10.1042/BJ20101771

[43] Campbell KP et al (1983) Purification and characterization of calsequestrin from canine cardiac sarcoplasmic reticulum and identification of the 53,000 Dalton glycoprotein. J Biol Chem 258(2):1197–1204 6337133

[44] Manno C et al (2017) Calsequestrin depolymerizes when calcium is depleted in the sarcoplasmic reticulum of working muscle. Proc Natl Acad Sci U S A 114(4):E638–e647 28069951 10.1073/pnas.1620265114 PMC5278470

[45] Beard NA, Laver DR, Dulhunty AF (2004) Calsequestrin and the calcium release channel of skeletal and cardiac muscle. Prog Biophys Mol Biol 85(1):33–69 15050380 10.1016/j.pbiomolbio.2003.07.001

[46] Wei L et al (2009) Unique isoform-specific properties of calsequestrin in the heart and skeletal muscle. Cell Calcium 45(5):474–484 19376574 10.1016/j.ceca.2009.03.006

[47] Zhao X et al (2010) Increased store-operated Ca2 + entry in skeletal muscle with reduced calsequestrin-1 expression. Biophys J 99(5):1556–1564 20816068 10.1016/j.bpj.2010.06.050 PMC2931717

[48] Marchena M et al (2020) Buffering and total calcium levels determine the presence of oscillatory regimes in cardiac cells. PLoS Comput Biol 16(9):e1007728 32970668 10.1371/journal.pcbi.1007728 PMC7537911

[49] Bers DM, Eisner DA, Valdivia HH (2003) Sarcoplasmic Reticulum Ca 2 Heart Fail Roles Diastolic Leak Ca 2 Transp 10.1161/01.RES.0000091871.54907.6B 14500331

[50] Györke I et al (2004) The role of calsequestrin, triadin, and junctin in conferring cardiac Ryanodine receptor responsiveness to luminal calcium. Biophys J 86(4):2121–2128 15041652 10.1016/S0006-3495(04)74271-X PMC1304063

[51] Qin J et al (2008) Luminal Ca2 + regulation of single cardiac Ryanodine receptors: insights provided by calsequestrin and its mutants. J Gen Physiol 131(4):325–334 18347081 10.1085/jgp.200709907 PMC2279168

[52] Györke S, Stevens SC, Terentyev D (2009) Cardiac calsequestrin: quest inside the SR. J Physiol 587(Pt 13):3091–3094 19567748 10.1113/jphysiol.2009.172049 PMC2727018

[53] Beard NA et al (2005) Regulation of Ryanodine receptors by calsequestrin: effect of high luminal Ca2 + and phosphorylation. Biophys J 88(5):3444–3454 15731387 10.1529/biophysj.104.051441 PMC1305491

[54] Bal NC et al (2010) The catecholaminergic polymorphic ventricular tachycardia mutation R33Q disrupts the N-terminal structural motif that regulates reversible calsequestrin polymerization. J Biol Chem 285(22):17188–17196 20353949 10.1074/jbc.M109.096354 PMC2878038

[55] Cerrone M et al (2007) Arrhythmogenic mechanisms in a mouse model of catecholaminergic polymorphic ventricular tachycardia. Circ Res 101(10):1039–1048 17872467 10.1161/CIRCRESAHA.107.148064 PMC2515360

[56] Rizzi N et al (2008) Unexpected structural and functional consequences of the R33Q homozygous mutation in cardiac calsequestrin: a complex arrhythmogenic cascade in a knock in mouse model. Circ Res 103(3):298–306 18583715 10.1161/CIRCRESAHA.108.171660

[57] Soeller C et al (2007) Analysis of Ryanodine receptor clusters in rat and human cardiac myocytes. Proc Natl Acad Sci U S A 104(38):14958–14963 17848521 10.1073/pnas.0703016104 PMC1986595

[58] Fabiato A (1983) Calcium-induced release of calcium from the cardiac sarcoplasmic reticulum. Am J Physiol 245(1):C1–14 6346892 10.1152/ajpcell.1983.245.1.C1

[59] Priori SG et al (2002) Clinical and molecular characterization of patients with catecholaminergic polymorphic ventricular tachycardia. Circulation 106(1):69–74 12093772 10.1161/01.cir.0000020013.73106.d8

[60] Knollmann BC (2009) New roles of calsequestrin and triadin in cardiac muscle. J Physiol 587(Pt 13):3081–3087 19451205 10.1113/jphysiol.2009.172098 PMC2727016

[61] Terentyev D et al (2007) Protein protein interactions between Triadin and calsequestrin are involved in modulation of sarcoplasmic reticulum calcium release in cardiac myocytes. J Physiol 583(Pt 1):71–80 17569730 10.1113/jphysiol.2007.136879 PMC2277233

[62] Aronsen JM, Swift F, Sejersted OM (2013) Cardiac sodium transport and excitation-contraction coupling. J Mol Cell Cardiol 61:11–19 23774049 10.1016/j.yjmcc.2013.06.003

[63] Murphy RM et al (2011) Quantification of calsequestrin 2 (CSQ2) in sheep cardiac muscle and Ca2+-binding protein changes in CSQ2 knockout mice. Am J Physiol Heart Circ Physiol 300(2):H595–604 21131479 10.1152/ajpheart.00902.2010 PMC3044055

[64] Györke S et al (2002) Regulation of sarcoplasmic reticulum calcium release by luminal calcium in cardiac muscle. Front Biosci 7:d1454–d1463 12045014 10.2741/A852

[65] Michelucci A et al (2018) Role of STIM1/ORAI1-mediated store-operated Ca(2+) entry in skeletal muscle physiology and disease. Cell Calcium 76:101–115 30414508 10.1016/j.ceca.2018.10.004 PMC6290926

[66] Wang L et al (2015) Retrograde regulation of STIM1-Orai1 interaction and store-operated Ca2 + entry by calsequestrin. Sci Rep 5:11349 26087026 10.1038/srep11349 PMC4471903

[67] Rosenberg P, Katz D, Bryson V (2019) SOCE and STIM1 signaling in the heart: timing and location matter. Cell Calcium 77:20–28 30508734 10.1016/j.ceca.2018.11.008 PMC9520449

[68] Baksh S et al (1995) Zn2 + binding to cardiac calsequestrin. Biochem Biophys Res Commun 209(1):310–315 7726852 10.1006/bbrc.1995.1504

[69] Carreras-Sureda A et al (2019) Non-canonical function of IRE1α determines mitochondria-associated Endoplasmic reticulum composition to control calcium transfer and bioenergetics. Nat Cell Biol 21(6):755–767 31110288 10.1038/s41556-019-0329-y PMC7246037

[70] Ackerman MJ et al (2011) HRS/EHRA expert consensus statement on the state of genetic testing for the channelopathies and cardiomyopathies this document was developed as a partnership between the heart rhythm society (HRS) and the European heart rhythm association (EHRA). Heart Rhythm 8(8):1308–1339 21787999 10.1016/j.hrthm.2011.05.020

[71] Groenendyk J et al (2010) Biology of Endoplasmic reticulum stress in the heart. Circ Res 107(10):1185–1197 21071716 10.1161/CIRCRESAHA.110.227033

[72] Belevych AE et al (2011) The relationship between arrhythmogenesis and impaired contractility in heart failure: role of altered Ryanodine receptor function. Cardiovasc Res 90(3):493–502 21273243 10.1093/cvr/cvr025 PMC3096306

[73] Loescher CM, Gibson LM, Stephenson DG (2019) Dantrolene sodium increases calcium binding by human Recombinant cardiac calsequestrin and calcium loading by sheep cardiac sarcoplasmic reticulum. Acta Physiol (Oxf) 226(3):e13261 30710413 10.1111/apha.13261

[74] Glukhov AV et al (2015) Calsequestrin 2 deletion causes sinoatrial node dysfunction and atrial arrhythmias associated with altered sarcoplasmic reticulum calcium cycling and degenerative fibrosis within the mouse atrial pacemaker complex1. Eur Heart J 36(11):686–697 24216388 10.1093/eurheartj/eht452 PMC4359358

[75] Denegri M et al (2014) Single delivery of an adeno-associated viral construct to transfer the CASQ2 gene to knock-in mice affected by catecholaminergic polymorphic ventricular tachycardia is able to cure the disease from birth to advanced age. Circulation 129(25):2673–2681 24888331 10.1161/CIRCULATIONAHA.113.006901

[76] Hudson MB, Price SR (2013) Calcineurin: a poorly understood regulator of muscle mass. Int J Biochem Cell Biol 45(10):2173–2178 23838168 10.1016/j.biocel.2013.06.029 PMC3947871

[77] Fisher DJ, Tate CA, Phillips S (1992) Developmental regulation of the sarcoplasmic reticulum calcium pump in the rabbit heart. Pediatr Res 31(5):474–479 1318540 10.1203/00006450-199205000-00012

[78] Meissner JD et al (2007) Activation of the beta myosin heavy chain promoter by MEF-2D, MyoD, p300, and the calcineurin/NFATc1 pathway. J Cell Physiol 211(1):138–148 17111365 10.1002/jcp.20916

[79] Song L et al (2007) Calsequestrin 2 (CASQ2) mutations increase expression of calreticulin and Ryanodine receptors, causing catecholaminergic polymorphic ventricular tachycardia. J Clin Invest 117(7):1814–1823 17607358 10.1172/JCI31080 PMC1904315

[80] Venetucci LA et al (2008) The sarcoplasmic reticulum and arrhythmogenic calcium release. Cardiovasc Res 77(2):285–292 18006483 10.1093/cvr/cvm009

[81] Sanchez EJ et al (2011) Phosphorylation of human calsequestrin: implications for calcium regulation. Mol Cell Biochem 353(1):195–204 21416293 10.1007/s11010-011-0787-4

[82] Ram ML et al (2004) Phosphorylation and dephosphorylation of calsequestrin on CK2-sensitive sites in heart. Mol Cell Biochem 266(1–2):209–217 15646044 10.1023/b:mcbi.0000049164.28580.56

[83] McFarland TP, Milstein ML, Cala SE (2010) Rough endoplasmic reticulum to junctional sarcoplasmic reticulum trafficking of calsequestrin in adult cardiomyocytes. J Mol Cell Cardiol 49(4):556–564 20595002 10.1016/j.yjmcc.2010.05.012 PMC2932759

[84] Tagliabracci VS et al (2015) A single kinase generates the majority of the secreted phosphoproteome. Cell 161(7):1619–1632 26091039 10.1016/j.cell.2015.05.028 PMC4963185

[85] Harada Y et al (2019) Oligosaccharyltransferase: A gatekeeper of health and tumor progression. Int J Mol Sci 20(23):6074 31810196 10.3390/ijms20236074 PMC6929149

[86] O’Brian JJ et al (2002) Mass spectrometry of cardiac calsequestrin characterizes microheterogeneity unique to heart and indicative of complex intracellular transit. J Biol Chem 277(40):37154–37160 12147690 10.1074/jbc.M204370200

[87] Milstein ML, Houle TD, Cala SE (2009) Calsequestrin isoforms localize to different ER subcompartments: evidence for polymer and heteropolymer-dependent localization. Exp Cell Res 315(3):523–534 19059396 10.1016/j.yexcr.2008.11.006

[88] Sanchez EJ et al (2012) Glycosylation of skeletal calsequestrin: implications for its function. J Biol Chem 287(5):3042–3050 22170046 10.1074/jbc.M111.326363 PMC3270961

[89] Zarain-Herzberg A, Estrada-Avilés R, Fragoso-Medina J (2012) Regulation of sarco(endo)plasmic reticulum Ca2+-ATPase and calsequestrin gene expression in the heart. Can J Physiol Pharmacol 90(8):1017–1028 22784385 10.1139/y2012-057

[90] Estrada-Avilés R, Rodríguez G, Zarain-Herzberg A (2017) The cardiac calsequestrin gene transcription is modulated at the promoter by NFAT and MEF-2 transcription factors. PLoS ONE 12(9):e0184724 28886186 10.1371/journal.pone.0184724 PMC5590987

[91] Reyes-Juárez JL et al (2007) Transcriptional analysis of the human cardiac calsequestrin gene in cardiac and skeletal Myocytes *. J Biol Chem 282(49):35554–35563 17938175 10.1074/jbc.M707788200

[92] Infante C, Ponce M, Manchado M (2011) Duplication of calsequestrin genes in teleosts: molecular characterization in the Senegalese sole (*Solea senegalensis*). Comp Biochem Physiol B: Biochem Mol Biol 158(4):304–314 21256971 10.1016/j.cbpb.2011.01.002

[93] Györke S, Terentyev D (2008) Modulation of ryanodine receptor by luminal calcium and accessory proteins in health and cardiac disease. Cardiovasc Res 77(2):245–255 18006456 10.1093/cvr/cvm038

[94] Fowler ED, Zissimopoulos S (2022) Molecular, subcellular, and arrhythmogenic mechanisms in genetic RyR2 disease. Biomolecules 12(8):1030 35892340 10.3390/biom12081030 PMC9394283

[95] Shin DW, Ma J, Kim DH (2000) The asp-rich region at the carboxyl-terminus of calsequestrin binds to Ca2 + and interacts with triadin. FEBS Lett 486(2):178–182 11113462 10.1016/s0014-5793(00)02246-8

[96] Zhang L et al (1997) Complex formation between Junctin, Triadin, Calsequestrin, and the Ryanodine receptor: PROTEINS OF THE CARDIAC JUNCTIONAL SARCOPLASMIC RETICULUM MEMBRANE *. J Biol Chem 272(37):23389–23397 9287354 10.1074/jbc.272.37.23389

[97] Wu L et al (2020) SARS-CoV-2 and cardiovascular complications: from molecular mechanisms to pharmaceutical management. Biochem Pharmacol 178:114114 32579957 10.1016/j.bcp.2020.114114 PMC7306106

[98] O’Shea CJ et al (2021) Ventricular arrhythmia burden during the coronavirus disease 2019 (COVID-19) pandemic. Eur Heart J 42(5):520–528 33321517 10.1093/eurheartj/ehaa893 PMC7953962

[99] Turker I et al (2017) Drug-induced fatal arrhythmias: acquired long QT and Brugada syndromes. Pharmacol Ther 176:48–59 28527921 10.1016/j.pharmthera.2017.05.001

[100] Shah M, Akar FG, Tomaselli GF (2005) Molecular basis of arrhythmias. Circulation 112(16):2517–2529 16230503 10.1161/CIRCULATIONAHA.104.494476

[101] Koppikar S et al (2013) Stroke and ventricular arrhythmias. Int J Cardiol 168(2):653–659 23602297 10.1016/j.ijcard.2013.03.058

[102] Schwartz PJ et al (2020) Inherited cardiac arrhythmias. Nat Rev Dis Primers 6(1):58 32678103 10.1038/s41572-020-0188-7 PMC7935690

[103] Gandjbakhch E et al (2018) Clinical Diagnosis, Imaging, and genetics of arrhythmogenic right ventricular Cardiomyopathy/Dysplasia: JACC State-of-the-Art review. J Am Coll Cardiol 72(7):784–804 30092956 10.1016/j.jacc.2018.05.065

[104] Haïssaguerre M et al (2020) Idiopathic ventricular fibrillation: role of purkinje system and microstructural myocardial abnormalities. JACC Clin Electrophysiol 6(6):591–608 32553208 10.1016/j.jacep.2020.03.010 PMC7308805

[105] Landstrom AP, Dobrev D, Wehrens XHT (2017) Calcium signaling and cardiac arrhythmias. Circ Res 120(12):1969–1993 28596175 10.1161/CIRCRESAHA.117.310083 PMC5607780

[106] Carlo Napolitano AM, Bloise R, Silvia G, Priori (2022) Catecholaminergic polymorphic ventricular tachycardia. University of Washington, Seattle 20301466

[107] Priori SG et al (2021) Precision medicine in catecholaminergic polymorphic ventricular tachycardia: JACC focus seminar 5/5. J Am Coll Cardiol 77(20):2592–2612 34016269 10.1016/j.jacc.2020.12.073

[108] Wang Q et al (2020) Phylogenetic and biochemical analysis of calsequestrin structure and association of its variants with cardiac disorders. Sci Rep 10(1):18115 33093545 10.1038/s41598-020-75097-3 PMC7582152

[109] Valle G et al (2014) Post-natal heart adaptation in a knock-in mouse model of calsequestrin 2-linked recessive catecholaminergic polymorphic ventricular tachycardia. Exp Cell Res 321(2):178–189 24370574 10.1016/j.yexcr.2013.12.014

[110] Valle G, Arad M, Volpe P (2020) Molecular adaptation to calsequestrin 2 (CASQ2) point mutations leading to catecholaminergic polymorphic ventricular tachycardia (CPVT): comparative analysis of R33Q and D307H mutants. J Muscle Res Cell Motil 41(2–3):251–258 32902830 10.1007/s10974-020-09587-2 PMC7666291

[111] Offerhaus JA, Bezzina CR, Wilde AAM (2020) Epidemiology of inherited arrhythmias. Nat Rev Cardiol 17(4):205–215 31582838 10.1038/s41569-019-0266-2

[112] Song J et al (2021) Advances in the molecular genetics of catecholaminergic polymorphic ventricular tachycardia. Front Pharmacol 12:718208 34483927 10.3389/fphar.2021.718208 PMC8415552

[113] Priori SG et al (2013) HRS/EHRA/APHRS expert consensus statement on the diagnosis and management of patients with inherited primary arrhythmia syndromes: document endorsed by HRS, EHRA, and APHRS in May 2013 and by ACCF, AHA, PACES, and AEPC in June 2013. Heart Rhythm 10(12):1932–1963 24011539 10.1016/j.hrthm.2013.05.014

[114] Kallas D et al (2021) Pediatric catecholaminergic polymorphic ventricular tachycardia: a translational perspective for the clinician-scientist. Int J Mol Sci. 10.3390/ijms22179293 34502196 10.3390/ijms22179293 PMC8431429

[115] Kawata H et al (2016) Catecholaminergic polymorphic ventricular tachycardia (CPVT) associated with Ryanodine receptor (RyR2) gene Mutations - Long-Term prognosis after initiation of medical treatment. Circ J 80(9):1907–1915 27452199 10.1253/circj.CJ-16-0250

[116] Kulbachinskaya E, Bereznitskaya V (2024) Long-term clinical course of patients with catecholaminergic polymorphic ventricular tachycardia: a more than 10-year follow-up cohort study. Ann Pediatr Cardiol 17(3):196–203 39564160 10.4103/apc.apc_101_24 PMC11573199

[117] Lahat H, Pras E, Eldar M (2002) Autosomal recessive catecholamine-induced polymorphic ventricular tachycardia. Exp Clin Cardiol 7(2–3):128–130 19649236 PMC2719177

[118] Postma AV et al (2002) Absence of calsequestrin 2 causes severe forms of catecholaminergic polymorphic ventricular tachycardia. Circ Res 91(8):e21–e26 12386154 10.1161/01.res.0000038886.18992.6b

[119] Houle TD, Ram ML, Cala SE (2004) Calsequestrin mutant D307H exhibits depressed binding to its protein targets and a depressed response to calcium. Cardiovasc Res 64(2):227–233 15485681 10.1016/j.cardiores.2004.09.009

[120] Lahat H et al (2001) A missense mutation in a highly conserved region of CASQ2 is associated with autosomal recessive Catecholamine-Induced polymorphic ventricular tachycardia in bedouin families from israel. Am J Hum Genet 69(6):1378–1384 11704930 10.1086/324565 PMC1235548

[121] Kalyanasundaram A et al (2010) The calsequestrin mutation CASQ2 < sup > D307H Does not affect protein stability and targeting to the junctional sarcoplasmic reticulum but compromises its dynamic regulation of calcium Buffering *. J Biol Chem 285(5):3076–3083 19920148 10.1074/jbc.M109.053892 PMC2823443

[122] Dirksen WP et al (2007) A mutation in calsequestrin, CASQ2D307H, impairs sarcoplasmic reticulum Ca2 + handling and causes complex ventricular arrhythmias in mice. Cardiovascular Res 75(1):69–78 10.1016/j.cardiores.2007.03.002 PMC2717009 17449018

[123] Viatchenko-Karpinski S et al (2004) Abnormal calcium signaling and sudden cardiac death associated with mutation of calsequestrin. Circul Res 94(4):471–477 10.1161/01.RES.0000115944.10681.EB 14715535

[124] Terentyev D et al (2006) Abnormal interactions of calsequestrin with the Ryanodine receptor calcium release channel complex linked to exercise-induced sudden cardiac death. Circ Res 98(9):1151–1158 16601229 10.1161/01.RES.0000220647.93982.08

[125] Ng K et al (2020) An international multicenter evaluation of inheritance Patterns, arrhythmic Risks, and underlying mechanisms of CASQ2-Catecholaminergic polymorphic ventricular tachycardia. Circulation 142(10):932–947 32693635 10.1161/CIRCULATIONAHA.120.045723 PMC7484339

[126] Zhang J-c et al (2018) Calcium-Mediated Oscillation in membrane potentials and atrial-Triggered activity in atrial cells of Casq2R33Q/R33Q mutation mice. Frontiers in Physiology, pp 9–2018 10.3389/fphys.2018.01447 PMC6224359 30450052

[127] Terentyev D et al (2008) Modulation of SR Ca release by luminal Ca and calsequestrin in cardiac myocytes: effects of CASQ2 mutations linked to sudden cardiac death. Biophys J 95(4):2037–2048 18469084 10.1529/biophysj.107.128249 PMC2483765

[128] Li Q et al (2019) CASQ2 variants in Chinese children with catecholaminergic polymorphic ventricular tachycardia. Mol Genet Genom Med 7(11):e949 10.1002/mgg3.949 PMC6825949 31482657

[129] Valle G et al (2008) Catecholaminergic polymorphic ventricular tachycardia-related mutations R33Q and L167H alter calcium sensitivity of human cardiac calsequestrin. Biochem J 413(2):291–303 18399795 10.1042/BJ20080163

[130] di Barletta MR et al (2006) Clinical phenotype and functional characterization of CASQ2 mutations associated with catecholaminergic polymorphic ventricular tachycardia. Circulation 114(10):1012–1019 16908766 10.1161/CIRCULATIONAHA.106.623793

[131] de la Fuente S et al (2008) A case of catecholaminergic polymorphic ventricular tachycardia caused by two calsequestrin 2 mutations. Pacing Clin Electrophysiol 31(7):916–919 18684293 10.1111/j.1540-8159.2008.01111.x

[132] Wong CH et al (2009) Genetic variability of RyR2 and CASQ2 genes in an Asian population. Forensic Sci Int 192(1–3):53–55 19709828 10.1016/j.forsciint.2009.07.019

[133] Laitinen PJ, Swan H, Kontula K (2003) Molecular genetics of exercise-induced polymorphic ventricular tachycardia: identification of three novel cardiac ryanodine receptor mutations and two common calsequestrin 2 amino-acid polymorphisms. Eur J Hum Genet 11(11):888–891 14571276 10.1038/sj.ejhg.5201061

[134] Lin D-J et al (2021) The link between abnormalities of calcium handling proteins and catecholaminergic polymorphic ventricular tachycardia. Tzu Chi Med J. 10.4103/tcmj.tcmj_288_20 34760626 10.4103/tcmj.tcmj_288_20 PMC8532576

[135] Kirchhefer U et al (2010) The human CASQ2 mutation K206N is associated with hyperglycosylation and altered cellular calcium handling. J Mol Cell Cardiol 49(1):95–105 20302875 10.1016/j.yjmcc.2010.03.006

[136] Fujisawa T et al (2019) A homozygous CASQ2 mutation in a Japanese patient with catecholaminergic polymorphic ventricular tachycardia, vol 2019. Case Reports in Genetics, p 9056596. 1 10.1155/2019/9056596 PMC6341267 30729048

[137] Liu QQ et al (2008) [A novel mutation of F189L in CASQ2 in families with catecholaminergic polymorphic ventricular tachycardia]. Zhonghua Yi Xue Yi Chuan Xue Za Zhi 25(3):334–337 18543230

[138] Rajagopalan A, Pollanen MS (2016) Sudden death during struggle in the setting of heterozygosity for a mutation in Calsequesterin 2. Forensic Sci Med Pathol 12(1):86–89 26671417 10.1007/s12024-015-9733-1

[139] Eckey K et al (2010) Modulation of human ether A Gogo related channels by CASQ2 contributes to etiology of catecholaminergic polymorphic ventricular tachycardia (CPVT). Cell Physiol Biochem 26(4–5):503–512 21063088 10.1159/000322318

[140] HONG RA et al (2012) Flecainide suppresses Defibrillator-Induced storming in catecholaminergic polymorphic ventricular tachycardia. Pacing Clin Electrophysiol 35(7):794–797 22553997 10.1111/j.1540-8159.2012.03421.x

[141] Josephs K et al (2017) Compound heterozygous CASQ2 mutations and long-term course of catecholaminergic polymorphic ventricular tachycardia. Mol Genet Genom Med 5(6):788–794 10.1002/mgg3.323 PMC5702571 29178653

[142] Gray B et al (2016) A novel heterozygous mutation in cardiac calsequestrin causes autosomal dominant catecholaminergic polymorphic ventricular tachycardia. Heart Rhythm 13(8):1652–1660 27157848 10.1016/j.hrthm.2016.05.004 PMC5453511

[143] Sy RW et al (2011) Arrhythmia characterization and long-term outcomes in catecholaminergic polymorphic ventricular tachycardia. Heart Rhythm 8(6):864–871 21315846 10.1016/j.hrthm.2011.01.048

[144] Steinberg C et al (2023) Ryr2-ryanodinopathies: from calcium overload to calcium deficiency. Europace. 10.1093/europace/euad156 37387319 10.1093/europace/euad156 PMC10311407

[145] Sumitomo N et al (2007) Association of atrial arrhythmia and sinus node dysfunction in patients with catecholaminergic polymorphic ventricular tachycardia. Circ J 71(10):1606–1609 17895559 10.1253/circj.71.1606

[146] van der Werf C et al (2012) Familial evaluation in catecholaminergic polymorphic ventricular tachycardia: disease penetrance and expression in cardiac Ryanodine receptor mutation-carrying relatives. Circ Arrhythm Electrophysiol 5(4):748–756 22787013 10.1161/CIRCEP.112.970517

[147] Wallace MJ et al (2021) Genetic complexity of sinoatrial node dysfunction. Front Genet 12:654925 33868385 10.3389/fgene.2021.654925 PMC8047474

[148] Priori SG et al (2015) ESC Guidelines for the management of patients with ventricular arrhythmias and the prevention of sudden cardiac death: The task force for the management of patients with ventricular arrhythmias and the prevention of sudden cardiac death of the European Society of Cardiology (ESC). Endorsed by: Association for European Paediatric and Congenital Cardiology (AEPC*).* Eur Heart J, 2015. 36(41): pp. 2793–2867 10.1093/eurheartj/ehv316 26320108

[149] Hayashi M et al (2009) Incidence and risk factors of arrhythmic events in catecholaminergic polymorphic ventricular tachycardia. Circulation 119(18):2426–2434 19398665 10.1161/CIRCULATIONAHA.108.829267

[150] Leren IS et al (2016) Nadolol decreases the incidence and severity of ventricular arrhythmias during exercise stress testing compared with β1-selective β-blockers in patients with catecholaminergic polymorphic ventricular tachycardia. Heart Rhythm 13(2):433–440 26432584 10.1016/j.hrthm.2015.09.029

[151] Salvage SC et al (2022) How does flecainide impact RyR2 channel function? J Gen Physiol. 10.1085/jgp.202213089 35713932 10.1085/jgp.202213089 PMC9208819

[152] Kramer CC et al (2020) Flipping syncope: the case of an adolescent athlete with syncopal episodes ultimately diagnosed with catecholaminergic polymorphic ventricular tachycardia. Clin Case Rep 8(8):1409–1412 32884764 10.1002/ccr3.2854 PMC7455425

[153] Lee HL et al (2025) Mechanistic insights into melatonin’s antiarrhythmic effects in acute ischemia-reperfusion-injured rabbit hearts undergoing therapeutic hypothermia. Int J Mol Sci. 10.3390/ijms26020615 39859328 10.3390/ijms26020615 PMC11766167

[154] Lee HL et al (2024) Eleclazine suppresses ventricular fibrillation in failing rabbit hearts with ischemia-reperfusion injury undergoing therapeutic hypothermia. Pharmacology. 10.1159/000542292 39467530 10.1159/000542292 PMC12215167

[155] Casper EA et al (2025) Melatonin ameliorates inflammation and improves outcomes of ischemia/reperfusion injury in patients undergoing coronary artery bypass grafting surgery: a randomized placebo-controlled study. Apoptosis 30(1–2):267–281 39633112 10.1007/s10495-024-02040-6 PMC11799019

[156] Heidbuchel H et al (2021) Recommendations for participation in leisure-time physical activity and competitive sports in patients with arrhythmias and potentially arrhythmogenic conditions: part 1: supraventricular arrhythmias. A position statement of the section of sports cardiology and exercise from the European association of preventive cardiology (EAPC) and the European heart rhythm association (EHRA), both associations of the European society of cardiology. Eur J Prev Cardiol 28(14):1539–1551 32597206 10.1177/2047487320925635

[157] Collura CA et al (2009) Left cardiac sympathetic denervation for the treatment of long QT syndrome and catecholaminergic polymorphic ventricular tachycardia using video-assisted thoracic surgery. Heart Rhythm 6(6):752–759 19467503 10.1016/j.hrthm.2009.03.024

[158] Wilde AAM et al (2008) Left cardiac sympathetic denervation for catecholaminergic polymorphic ventricular tachycardia. N Engl J Med 358(19):2024–2029 18463378 10.1056/NEJMoa0708006

[159] Kaneko N et al (2009) Pharmacological characteristics and clinical applications of K201. Curr Clin Pharmacol 4(2):126–131 19442077 10.2174/157488409788184972 PMC2841427

[160] Wehrens XH et al (2004) Protection from cardiac arrhythmia through Ryanodine receptor-stabilizing protein calstabin2. Science 304(5668):292–296 15073377 10.1126/science.1094301

[161] Batiste SM et al (2019) Unnatural verticilide enantiomer inhibits type 2 Ryanodine receptor-mediated calcium leak and is antiarrhythmic. Proc Natl Acad Sci U S A 116(11):4810–4815 30792355 10.1073/pnas.1816685116 PMC6421472

[162] Penttinen K et al (2015) Antiarrhythmic effects of Dantrolene in patients with catecholaminergic polymorphic ventricular tachycardia and replication of the responses using iPSC models. PLoS ONE 10(5):e0125366 25955245 10.1371/journal.pone.0125366 PMC4425399

[163] Kurtzwald-Josefson E et al (2017) Viral delivered gene therapy to treat catecholaminergic polymorphic ventricular tachycardia (CPVT2) in mouse models. Heart Rhythm 14(7):1053–1060 28336343 10.1016/j.hrthm.2017.03.025

[164] Bezzerides VJ et al (2019) Gene therapy for catecholaminergic polymorphic ventricular tachycardia by Inhibition of Ca(2+)/Calmodulin-Dependent kinase II. Circulation 140(5):405–419 31155924 10.1161/CIRCULATIONAHA.118.038514 PMC7274838

[165] Jessup M et al (2011) Calcium upregulation by percutaneous administration of gene therapy in cardiac disease (CUPID): a phase 2 trial of intracoronary gene therapy of sarcoplasmic reticulum Ca2+-ATPase in patients with advanced heart failure. Circulation 124(3):304–313 21709064 10.1161/CIRCULATIONAHA.111.022889 PMC5843948

[166] Zsebo K et al (2014) Long-term effects of AAV1/SERCA2a gene transfer in patients with severe heart failure: analysis of recurrent cardiovascular events and mortality. Circ Res 114(1):101–108 24065463 10.1161/CIRCRESAHA.113.302421

[167] Bezzerides VJ et al (2020) Gene therapy for inherited arrhythmias. Cardiovasc Res 116(9):1635–1650 32321160 10.1093/cvr/cvaa107 PMC7341167

[168] Sasano T, Takahashi K, Sugiyama K (2013) Gene therapy for cardiac arrhythmias. Acta Cardiol Sin 29(3):226–234 27122711 PMC4804834

[169] Katz G et al (2010) Optimizing catecholaminergic polymorphic ventricular tachycardia therapy in calsequestrin-mutant mice. Heart Rhythm 7(11):1676–1682 20620233 10.1016/j.hrthm.2010.07.004 PMC4103178

[170] Lodola F et al (2016) Adeno-associated virus-mediated CASQ2 delivery rescues phenotypic alterations in a patient-specific model of recessive catecholaminergic polymorphic ventricular tachycardia. Cell Death Dis 7(10):e2393 27711080 10.1038/cddis.2016.304 PMC5133973

[171] Pölönen RP et al (2018) Antiarrhythmic effects of carvedilol and flecainide in cardiomyocytes derived from catecholaminergic polymorphic ventricular tachycardia patients. Stem Cells Int 2018:9109503 29760739 10.1155/2018/9109503 PMC5924967

[172] Ben Jehuda R, Barad L (2016) Patient specific induced pluripotent stem Cell-Derived cardiomyocytes for drug development and screening in catecholaminergic polymorphic ventricular tachycardia. J Atr Fibrillation 9(2):1423 27909533 10.4022/jafib.1423 PMC5129686

[173] Ranpura GN et al (2025) Generation of an isogenic CRISPR/Cas9-corrected control induced pluripotent stem cell line from a patient with autosomal dominant catecholaminergic polymorphic ventricular tachycardia with a heterozygous variant in cardiac calsequestrin-2. Stem Cell Res 83:103650 39826349 10.1016/j.scr.2024.103650

[174] Ran FA et al (2013) Genome engineering using the CRISPR-Cas9 system. Nat Protoc 8(11):2281–2308 24157548 10.1038/nprot.2013.143 PMC3969860

[175] Ross S et al (2019) Characterization of the first induced pluripotent stem cell line generated from a patient with autosomal dominant catecholaminergic polymorphic ventricular tachycardia due to a heterozygous mutation in cardiac calsequestrin-2. Stem Cell Res 37:101450 31039485 10.1016/j.scr.2019.101450

[176] Dridi H et al (2023) Heart failure-induced cognitive dysfunction is mediated by intracellular Ca(2+) leak through Ryanodine receptor type 2. Nat Neurosci 26(8):1365–1378 37429912 10.1038/s41593-023-01377-6 PMC10400432

[177] Liu Y et al (2023) Targeting Ryanodine receptor type 2 to mitigate chemotherapy-induced neurocognitive impairments in mice. Sci Transl Med 15(715):eadf8977 37756377 10.1126/scitranslmed.adf8977

[178] Du Y, Demillard LJ, Ren J (2022) Sarcoplasmic reticulum Ca(2+) dysregulation in the pathophysiology of inherited arrhythmia: an update. Biochem Pharmacol 200:115059 35490731 10.1016/j.bcp.2022.115059

[179] Marabelli C, Santiago DJ, Priori SG (2023) The structural-functional crosstalk of the calsequestrin system: insights and pathological implications. Biomolecules. 10.3390/biom13121693 38136565 10.3390/biom13121693 PMC10741413

[180] Henriquez E et al (2023) Catecholaminergic polymorphic ventricular tachycardia and gene therapy: A comprehensive review of the literature. Cureus 15(10):e47974 38034271 10.7759/cureus.47974 PMC10686237

